# Magnesium Nanoparticles for Surface-Enhanced Raman Scattering and Plasmon-Driven Catalysis

**DOI:** 10.1021/acsnano.4c06858

**Published:** 2024-07-04

**Authors:** Andrey Ten, Vladimir Lomonosov, Christina Boukouvala, Emilie Ringe

**Affiliations:** † Department of Materials Science and Metallurgy, University of Cambridge, 27 Charles Babbage Road, Cambridge CB3 0FS, United Kingdom; ‡ Department of Earth Sciences, University of Cambridge, Downing Street, Cambridge CB2 3EQ, United Kingdom

**Keywords:** Magnesium nanoparticles, Nanoplasmonics, LSPRs, SERS, Plasmon-enhanced catalysis

## Abstract

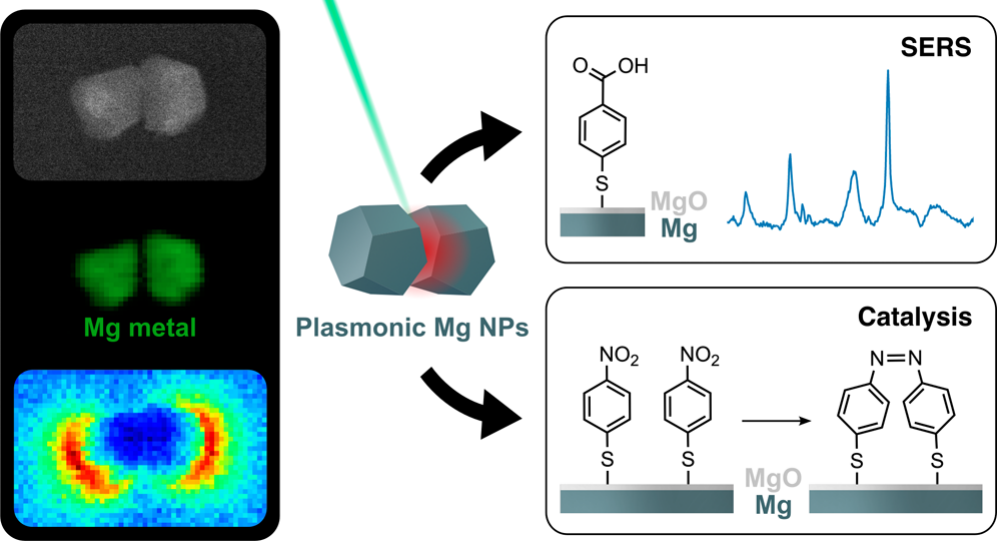

Nanostructures of some metals can sustain localized surface plasmon resonances, collective oscillations of free electrons excited by incident light. This effect results in wavelength-dependent absorption and scattering, enhancement of the incident electric field at the metal surface, and generation of hot carriers as a decay product. The enhanced electric field can be utilized to amplify the spectroscopic signal in surface-enhanced Raman scattering (SERS), while hot carriers can be exploited for catalytic applications. In recent years, cheaper and more earth abundant alternatives to traditional plasmonic Au and Ag have gained growing attention. Here, we demonstrate the ability of plasmonic Mg nanoparticles to enhance Raman scattering and drive chemical transformations upon laser irradiation. The plasmonic properties of Mg nanoparticles are characterized at the bulk and single particle level by optical spectroscopy and scanning transmission electron microscopy coupled with electron energy-loss spectroscopy and supported by numerical simulations. SERS enhancement factors of ∼10^2^ at 532 and 633 nm are obtained using 4-mercaptobenzoic acid and 4-nitrobenzenethiol. Furthermore, the reductive coupling of 4-nitrobenzenethiol to 4,4′-dimercaptoazobenzene is observed on the surface of Mg nanoparticles under 532 nm excitation in the absence of reducing agents, indicating a plasmon-driven catalytic process. Once decorated with Pd, Mg nanostructures display an enhancement factor of 10^3^ along with an increase in the rate of catalytic coupling. The results of this study demonstrate the successful application of plasmonic Mg nanoparticles in sensing and plasmon-enhanced catalysis.

Plasmonic metallic nanoparticles (NPs) can sustain localized surface plasmon resonances (LSPRs), coherent oscillations of conduction electrons. LSPRs are driven by an incident oscillating electric field, i.e., light, and lead to enhanced, wavelength-dependent absorption, scattering, and local field enhancement. The latter is at the basis of the signal amplification in surface-enhanced Raman scattering (SERS)^1,2^ and metal-enhanced fluorescence (MEF),^3,4^ for instance. While conventional Raman scattering suffers from an inherently low cross-section, plasmonic effects can generate enhancement factors (EFs) of several orders of magnitude, enabling few or even single molecule sensitivity.^5−7^ Typical SERS substrates are Au- and Ag-based,^8^ but recently, alternative plasmonic metals such as Cu^9^ and Al^10^ have gained attention for their different resonance ranges, biocompatibility, and lower cost than Au and Ag.^11^ Both Cu^12^ and Al^13^ have been explored as SERS substrates, reflecting the interest toward alternative materials. Mg is a biocompatible^14^ and earth-abundant metal, with a plasmonic quality factor superior to that of Au and Cu at wavelengths below 500 nm, and to that of Al across the visible range.^11^ Mg’s spontaneously formed oxide layer protects the metallic core against further oxidation by air and is not detrimental to its plasmonic properties;^15−17^ appropriate coatings can further provide protection against oxidation in aqueous medium.^18^ Despite these qualities and recent advances, SERS from Mg NPs has yet to be reported.

In addition to SERS effects, LSPRs can be utilized to drive chemical transformations on the surface of plasmonic NPs, owing to the coherent resonance decaying to hot carriers and eventually heat.^19−23^ This makes plasmonic NPs of interest for applications in light-enhanced catalysis. Examples of plasmon-mediated catalytic reactions include CO_2_ reduction,^24^ dry reforming of CH_4_,^25^ oxidation of NH_3_ and CO,^26^ C–F bond activation,^27^ and CH_3_OH decomposition.^28^ As plasmon-driven reactions occur on the surface of the metals supporting LSPRs, they can also be monitored by SERS. For instance, the decarboxylation of 4-mercaptobenzoic acid (4-MBA),^29,30^ and the reductive coupling of 4-nitrobenzenethiol (4-NBT) to 4,4′-dimercaptoazobenzene (DMAB)^31,32^ are common probes for examining a plasmonic substrate’s ability to drive chemical transformations.

Photocatalytic performance can be improved by decorating a plasmonic metal with small amounts of catalytically active albeit poorly plasmonic components.^33,34^ Cu and Al have been actively explored as inexpensive plasmonic cores for this application, incorporating catalytic metals such as Fe, Pd, Pt, Ru, and Rh.^25,33−37^ Such complexes demonstrated light-enhanced catalytic performance in a wide range of reactions including H_2_ dissociation and NH_3_ decomposition.^35,36^ Recently, the photocatalytic activity of plasmonic Mg-based complexes was demonstrated, with Mg–Pd NPs achieving a 2-fold decrease in the activation energy and enhanced selectivity under light excitation for acetylene hydrogenation.^38^ Further, the photocatalytic activity of bimetallic Mg–Au nanostructures in the reduction of 4-NBT to DMAB was demonstrated using tip-enhanced Raman spectroscopy (TERS),^39^ verifying the coupling between the Mg core and Au decorations.

Here, we demonstrate the application of Mg faceted spheroids as SERS substrates and SERS-trackable photocatalysts. We examine the SERS signal from 4-MBA and 4-NBT and calculate the EF for Mg NPs under 532 and 633 nm laser excitations. We show that the reductive coupling of 4-NBT to DMAB proceeds on the surface of Mg NPs protected by a native oxide layer under 532 nm laser excitation in the absence of additional catalytically active components and reducing agents. We further extend the investigation to Mg NPs decorated with Pd (Mg–Pd NPs) and compare their SERS signal, EF, and catalytic behavior to those of Mg NPs. We rationalize and support our SERS findings with experimental near-field studies with monochromated scanning transmission electron microscopy coupled with electron energy-loss spectroscopy (STEM-EELS) and numerical results obtained in the discrete-dipole approximation (DDA). This successful demonstration of SERS by Mg NPs unravels the potential of this earth abundant and biocompatible material for sensing applications, while the ability of Mg to drive surface reactions under light excitation further extends its application range in plasmon-enhanced catalysis.

## Results and Discussion

### Characterization of Mg NPs

**Figure 1:**
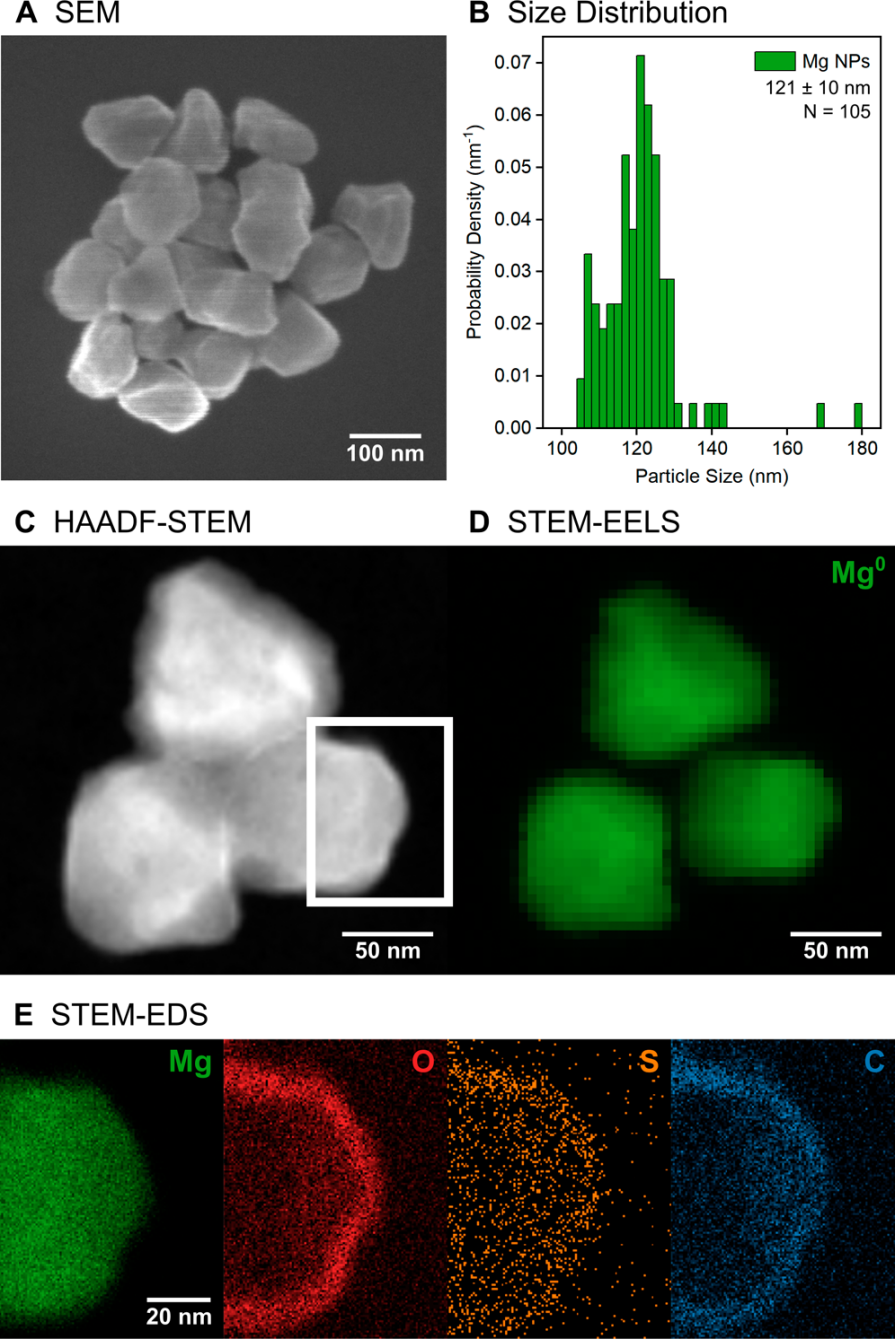
Metallic Mg faceted spheroids have a narrow size distribution and a thin oxide layer on which thiols bind. (A) SEM images and (B) size distribution histogram of as-synthesized Mg NPs. (C) HAADF-STEM image, (D) Mg bulk plasmon (∼10.1 eV) map indicating the distribution of metallic Mg, and (E) STEM-EDS elemental maps of Mg NPs after incubation with 4-MBA. The STEM-EDS maps were collected from the area marked by the white rectangle in the HAADF-STEM image.

Mg faceted spheroids (Figures 1A and S1) were synthesized colloidally following a recently published seed-mediated growth method.^40^ The resulting NPs are considerably more isotropic than the Mg hexagonal single crystal and twinned nanoplatelets previously studied optically.^15,41^ The average size of the long NP axis and its standard deviation, determined from scanning electron microscopy (SEM), are 121 ± 10 nm (Figure 1B). The extinction profile of a colloidal suspension of the NPs in isopropyl alcohol (IPA) displays a strong LSPR peak centered at 574 nm (Figure S2).

Colloidally synthesized Mg NPs have a native, self-limiting MgO layer that is protective in gas phase oxidative environments up to 400 °C^42^ but susceptible to water.^40,43^ The incubation of Mg NPs with the Raman reporter molecules 4-MBA and 4-NBT^44−48^ was thus performed in IPA to avoid degradation of Mg; ethanol and methanol could also be used but lead to poorer colloidal stability than IPA. NPs retain their metallic cores postincubation, as confirmed by the Mg bulk plasmon peak indicating metallic Mg, at ∼10.1 eV in STEM-EELS (Figures 1C,D and S3). No detectable change in the native oxide layer thickness was observed after the incubation. The oxide thickness of up to 10 nm, as mapped by STEM energy dispersive X-ray spectroscopy (STEM-EDS) maps (Figures 1E and S4), is in good agreement with that previously reported for as-synthesized Mg NPs.^15,49^ Note, this value is an upper bound as the analytes can contribute to the oxygen signal, and the oxide layer may appear thicker due to STEM projection effects from a faceted 3D object.

4-MBA and 4-NBT bind to the surface oxide of Mg NPs. The incubated Mg NPs were washed three times in IPA prior to STEM and SERS measurements. STEM-EDS maps reveal a localization of the S signal on the NPs (Figure 1E), and SERS signals from both analytes are observed (see next section). While thiols are not expected to interact in the same way with an MgO surface as in the known covalent Au–S bond,^44^ MgO has been studied for use as a desulfurizer to remove gaseous pollutants such as SO_2_ and H_2_S^50,51^ because it offers effective adsorption sites for those molecules at room temperature.^52−57^ H_2_S adsorbs to the Mg^2+^ sites in MgO through S.^52^ Analogously, 4-MBA and 4-NBT can be expected to bind to MgO via a deprotonated thiol formed in solution. Other interactions such as through the carboxyl group in 4-MBA and the nitro group in 4-NBT are possible, given that both acetic acid and NO_2_ can adsorb on MgO.^51,58^

### Binding and SERS Spectra of 4-MBA and 4-NBT on Mg NPs

**Figure 2:**
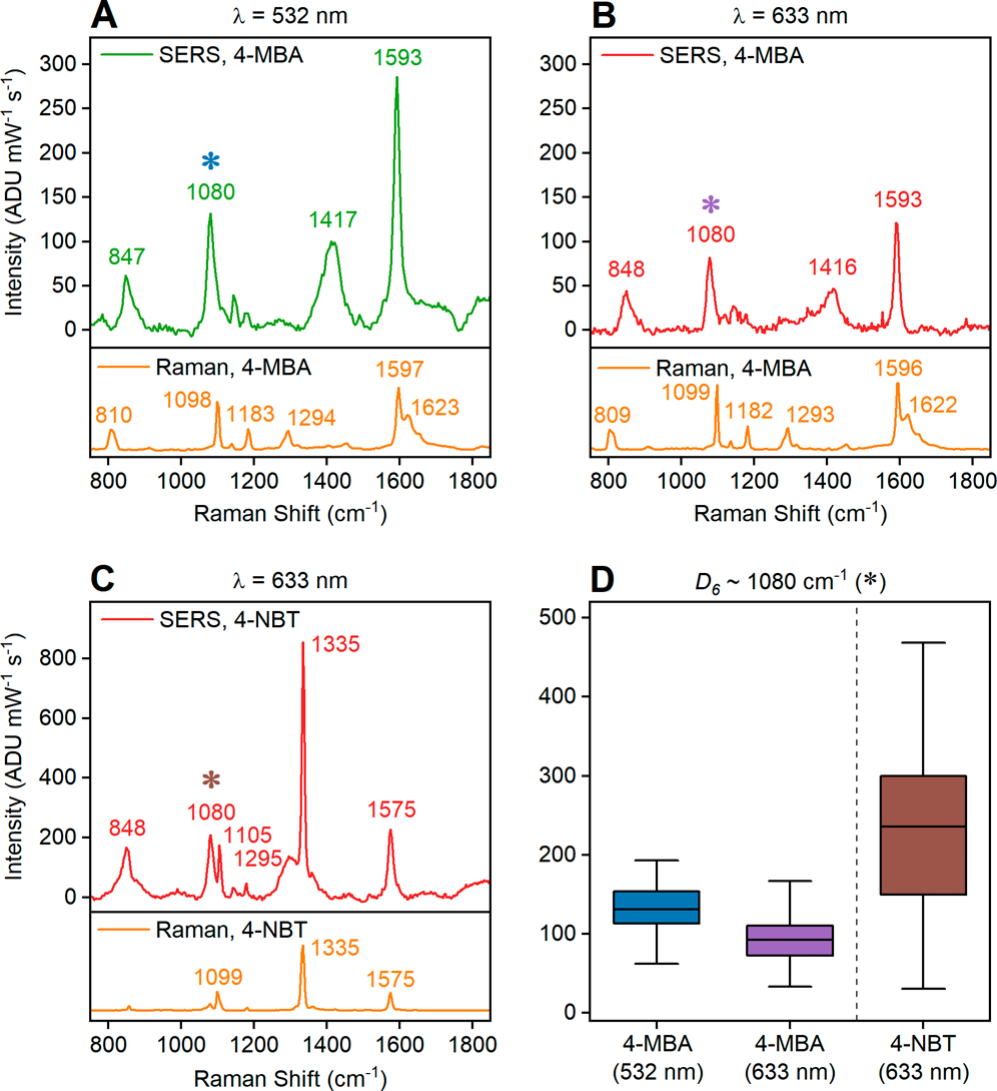
SERS spectra of analytes adsorbed on dry Mg NPs: (A) 4-MBA at 532 nm, (B) 4-MBA at 633 nm, and (C) 4-NBT at 633 nm. Normal Raman spectra of analytes in solid form at the same laser wavelength are shown under each SERS spectrum as a reference. (D) Variation of intensity of the peak at ∼1080 cm^–1^ (*D*_6_ mode, labeled with a colored asterisk in A–C) across 100 randomly selected regions. Full raw spectra without background subtraction are included in Figures S7 and S8.

Mg NPs enhance the Raman signal of surface-bound 4-MBA and 4-NBT at both 532 and 633 nm. SERS spectra were obtained from analyte-incubated colloidal Mg NPs deposited on membrane filters to produce dense regions of dry NPs (Figure S5).^59^ Differences observed when comparing analytes on dry NPs to their normal Raman spectra (Figure 2A–C) indicate a change in the analytes’ structure due to binding to the MgO surface. No Raman signals from either the membrane filter or MgO itself (Figure S6) are observed in the spectra.

With 4-MBA, 532 and 633 nm excitation give rise to identical spectral features, and no change to the spectra was observed over time, indicating the stability of the analyte on the surface of the NPs. The SERS signal intensity of the 1080 cm^–1^ peak of 100 randomly selected regions on dry Mg NPs is on average 42% higher at 532 nm compared to 633 nm (Figure 2D).

The 4-MBA SERS spectra are in good agreement with previous studies on Au and Ag,^45−48,60^ as well as on metal oxide semiconductor substrates.^61−63^ The position of SERS peaks indicates the presence of a deprotonated state (COO^–^), as discussed in detail in the Supporting Information (SI). Further, the absence of S–H stretching and bending modes near 2580 and 915 cm^–1^,^45,48^ respectively (Figure S7), implies dissociation of the S–H bond. Finally, the intense bands at 1080 and 1593 cm^–1^ in the SERS spectrum of 4-MBA arise from the aromatic ring^45−48,60,64^ and are, as described in the SI, assigned to *D*_6_ (containing C–S stretching) and *D*_3_ modes, respectively.^65^

The binding geometry of 4-MBA on the surface of the Mg NPs can be deduced from the SERS spectra. The carboxylate anion stretching mode near 1417 cm^–1^ indicates that COO^–^ is not involved in binding to Mg NPs, as otherwise the mode would appear at lower wavenumbers.^45,64^ Further, the absence of the out-of-plane ring vibration mode (estimated to be *D*_17_) near 718 cm^–1^ confirms that 4-MBA is not lying flat along the surface,^48,60,64^ and the presence of the *D*_1_ and *D*_2_ modes (containing aromatic C–H stretching) at 3063 cm^–1^ (Figure S7) indicates that 4-MBA is positioned perpendicular to the surface of Mg NPs with the carboxyl group pointing away from the surface.^64,66^ Therefore, 4-MBA molecules are coordinated to Mg NPs through the S atom, similar to Au and Ag.^64^

The SERS spectrum of 4-NBT at 633 nm also agrees with spectra on Au and Ag.^67−69,32,70^ As with 4-MBA, the 4-NBT S–H stretching mode at 2549 cm^–1^ in normal Raman is not present in SERS (Figures S7 and S8), implying binding through S^69^ (detailed mode assignments in the SI). At 532 nm, 4-NBT undergoes reductive coupling to DMAB. This reaction occurs much faster at 532 nm than at 633 nm on Ag NPs^71^ and only at 532 nm with Mg NPs; this catalytic coupling will be discussed later.

We also observe a mode in the 4-NBT SERS spectrum not commonly reported in the literature: a peak at 1295 cm^–1^. This shoulder is lower in energy than the N–O stretching mode and is attributed to the 4-NBT anion (4-NBT^–^). This feature has been reported with TERS, where the anion peak appeared at 1305 cm^–1^ on Au and 1289 cm^–1^ on Ag.^72,73^ Likewise, Choi et al. calculated that the anion radical of 4-NBT and its conjugate acid can induce a shift of the N–O stretching mode to below 1300 cm^–1^.^74^ The reduction of 4-NBT can, in principle, result in the formation of 4-aminobenzenethiol (4-ABT) or DMAB;^74−77^ however, neither is observed here, and we note that the 4-NBT spectrum remained unchanged over prolonged 633 nm excitation.

**Figure 3:**
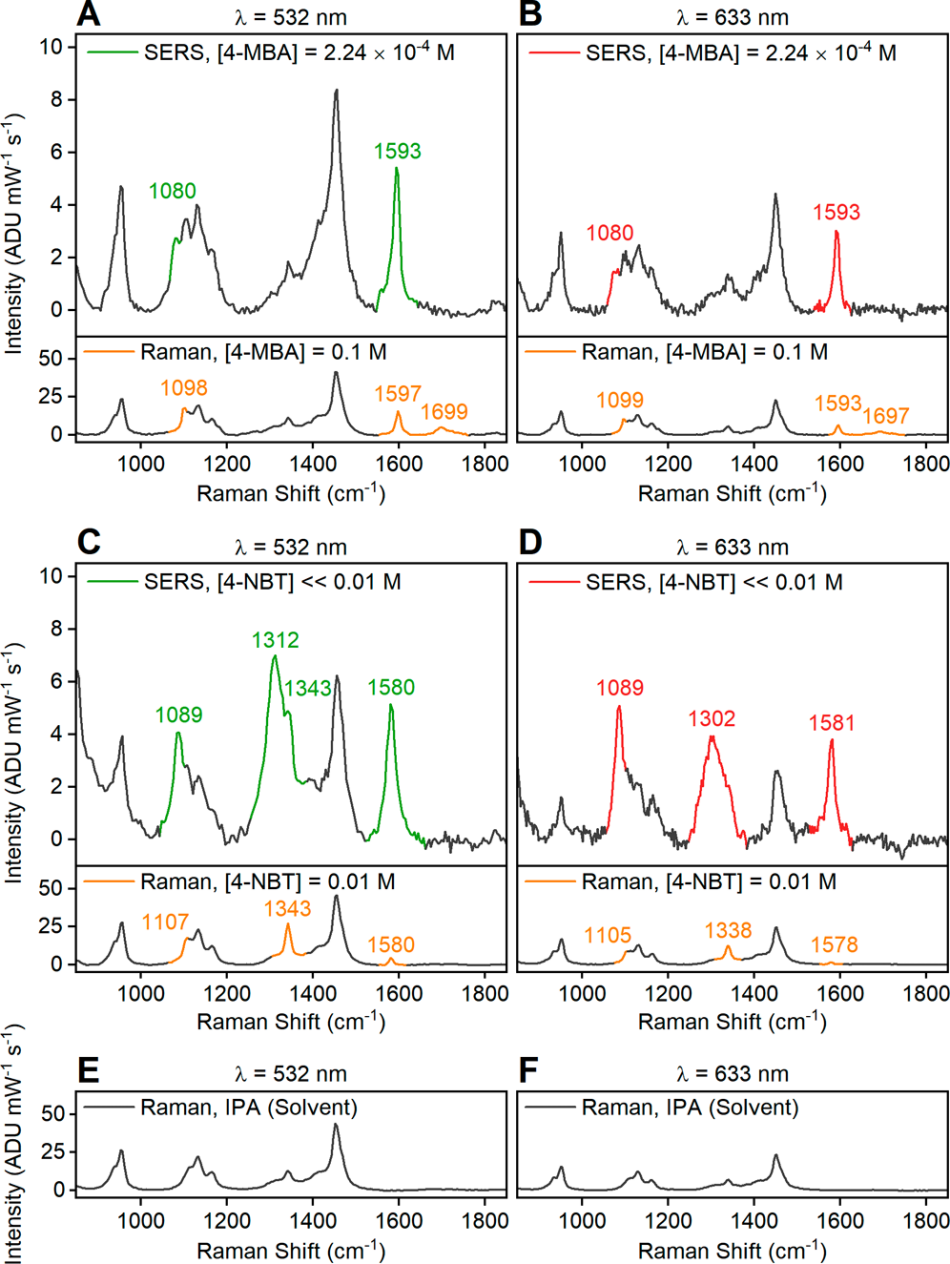
SERS spectra of analyte-incubated colloidal Mg NPs dispersed in IPA. The spectra were collected using 4-MBA at (A) 532 and (B) 633 nm and 4-NBT at (C) 532 and (D) 633 nm. The normal Raman spectra of 0.1 M 4-MBA solution and 0.01 M 4-NBT solution in IPA are shown as a reference, under each SERS spectra. The normal Raman spectra of IPA at (E) 532 and (F) 633 nm are presented as a reference. The spectral features of IPA are plotted in black in all plots while the peaks from analytes are highlighted in color. The 4-MBA concentration in SERS was quantified with ICP-OES (Table S1). The 4-NBT concentration in SERS was estimated from the incubation solution concentration and subsequent cleaning steps. Full raw spectra without background subtraction are reported in Figures S9–S11.

SERS is also observed for analyte-incubated Mg NPs dispersed in IPA. The spectra (Figure 3A–D, top; full spectra in Figures S9 and S10) include contributions from IPA (Figure 3E,F; full spectra in Figure S11), but the dominant analyte SERS peaks are clearly visible. In all cases, the *D*_3_ mode near 1580 cm^–1^ is spectrally distinct from the IPA Raman bands. Meanwhile, the normal Raman spectra of concentrated 4-MBA and 4-NBT (Figure 3A–D, bottom) solutions in IPA show the expected solvent peaks and unbound analyte peaks, which differ from those of SERS.

The peaks observed in the SERS spectra of 4-MBA on colloidal Mg NPs are consistent with those on dry NPs. Meanwhile, the SERS spectra of 4-NBT from colloidal Mg NPs are different from those of dry NPs. The most pronounced difference appears in the N–O stretching mode at 633 nm excitation. With colloidal NPs, a broad peak is observed at 1302 cm^–1^, shifted from the SERS peak on dry NPs (1335 cm^–1^) and the normal Raman peak (1338 cm^–1^). This shift to lower wavenumbers is attributed to the presence of 4-NBT^–^;^72,73^ the broadening indicates the coexistence of the neutral and anionic form. At 532 nm, this region displays an N–O stretching band at 1312 cm^–1^, indicative of 4-NBT^–^ and a shoulder at 1343 cm^–1^ from 4-NBT. The difference in the relative intensities of the N–O stretching modes of the anion and 4-NBT molecule at different wavelengths suggests that the incident electromagnetic radiation plays a role in the bond dissociation of 4-NBT on Mg NPs. The prominence of 4-NBT^–^ on colloidal NPs, compared to dry NPs, can be explained by its stabilization in IPA. The SERS spectrum of 4-NBT on colloidal NPs does not change over time, unlike that of dried NPs at 532 nm.

### EF of Mg NPs

Plasmonic effects largely contribute to the enhancement observed in SERS. Mg NPs comprise a metallic (confirmed by EELS, Figure 1) plasmonic core with a ∼10 nm oxide layer; we have previously demonstrated experimentally and numerically that this layer only minimally affects the LSPRs of Mg NPs.^15,41^ However, the oxide acts as a spacer separating the analyte molecules and the metal surface,^15,43,49^ such that a modest SERS EF is expected.

The colloidal SERS and normal Raman spectra are used to calculate the EF of Mg NPs using^78^
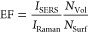
1where *I*_SERS_ and *I*_Raman_ are the intensities of a vibrational mode, and *N*_Surf_ and *N*_Vol_ are the number of analyte molecules probed in SERS and normal Raman, respectively. The intensities of the *D*_3_ mode at ∼1580 cm^–1^ are used for *I*_SERS_ and *I*_Raman_, as this peak does not overlap with any IPA Raman bands and is present for both analytes. *N*_Surf_ is calculated by estimating a monolayer coverage on the surface of NPs, an approach commonly used to calculate SERS EFs.^79,80^ We approximate the shape of a faceted spheroid as a sphere, e.g., a 121 nm diameter NP that has a surface area of 4.6 × 10^4^ nm^2^. Using 1.738 g cm^–3^ for the density of Mg,^14^ the concentration of Mg in colloids from ICP-OES analysis (Table S1), a thiol footprint of 0.22 nm^2^,^79^ and a scattering volume (volume of illumination) of 6.54 × 10^–17^ m^3^, *N*_Surf_ is calculated to be 1.09 × 10^7^ and 1.05 × 10^7^ molecules for 4-MBA and 4-NBT adsorbed on Mg NPs, respectively. *N*_Vol_ is calculated by multiplying the concentration of the analyte solution (0.1 M for 4-MBA and 0.01 M for 4-NBT) by the scattering volume.

The EFs for both analytes at 532 and 633 nm are on the order of 10^2^ (Table 1). To assess the validity of the monolayer surface coverage approximation, the S content was quantified by ICP-OES for the 4-MBA sample. The *N*_Surf_ obtained with this alternative method is 8.83 × 10^6^ molecules, consistent with the monolayer estimation (1.09 × 10^7^ molecules) and resulting in EFs of ∼10^2^ (Table S2). The EF of Mg NPs is higher at 633 nm, despite the SERS signal being higher at 532 nm in both dry and colloidal NPs. This effect occurs because the normal Raman signal, hence cross-section, is smaller for both analytes at 633 nm.

**Table 1:** EFs of Mg and Mg–Pd NPs Calculated Using *N*_Surf_ Obtained by the Monolayer Approximation

NPs	Mg				Mg–Pd			
Analyte	4-MBA		4-NBT		4-MBA		4-NBT	
Wavelength (nm)	532	633	532	633	532	633	532	633
*I*_SERS_ (ADU mW^–1^ s^–1^)	5.44	3.04	5.16	3.82	13.27	9.65	16.06	7.44
*I*_Raman_ (ADU mW^–1^ s^–1^)	15.80	6.19	4.44	1.74	7.19	2.45	3.74	1.39
*N*_Surf_ (molecules)	1.09 × 10^7^		1.05 × 10^7^		6.22 × 10^6^		7.92 × 10^6^	
*N*_Vol_ (molecules)	3.94 × 10^9^		3.94 × 10^8^		3.94 × 10^9^		3.94 × 10^8^	
EF	125	178	44	83	1167	2491	214	266

In comparison, a control experiment performed using 58 nm spherical Au NPs incubated with 4-MBA analyte under equivalent conditions leads to an EF of 10^2^ with monolayer approximation and 10^4^ when using ICP-OES of S content (Figure S12 and Table S3), confirming that the monolayer approach produces an underestimate of the EF.

The EFs reported in Table 1 are conservative estimates. First, the monolayer approximation and the ICP-OES of S both overestimate the number of analytes bound to the NP surface, with the former even more than the latter. Since the interaction of MgO with S is expected to be weaker than covalent binding between Au and S, the maximum packing can hardly be achieved on Mg NPs. Therefore, *N*_SERS_ is likely overestimated and EF underestimated. ICP-OES reports on all S-containing species, including those on the surface and in the solution. Using this concentration leads to the calculation of an upper bound for *N*_SERS_ and a lower bound for the EF. Further, eq 1 assumes that the scattering volume is identical between SERS and Raman measurements. Though the same acquisition conditions were used, the Mg NP colloids were opaque, while the analyte solutions were clear. This difference leads to a smaller effective scattering volume for the Mg-containing solution (SERS) and therefore a higher EF than what is calculated.

The enhancement from Mg NPs is not sufficient to observe SERS signal from single particles. Correlated dark field optical scattering spectroscopy, SERS, and SEM of Mg NPs (Figures S13 and S14) show that neither single particle nor small (<30 NPs) aggregates generate sufficient enhancement for signal detection. In addition, this correlation reveals the heterogeneity of random aggregates, both in size and arrangement, leading to a heterogeneous scattering response.

Finally, we argue that the enhancement obtained from Mg NPs is predominantly an electromagnetic effect. SERS mediated by a chemical enhancement mechanism alone would be independent of the NP shape. However, in this study, we do not observe SERS for Mg hexagonal nanoplatelets.^43^ As opposed to platelets, the formation of electromagnetic hot spots for spheroids is not sterically hindered, leading to a strong enhancement.

### Electromagnetic Localization and Enhancement by Mg NPs

SERS is commonly attributed to electromagnetic hot spots,^81^ such as those formed at the corners of sharp NPs and between NPs in aggregates. Here, we experimentally probed the hot spots formed around and between Mg faceted spheroids using monochromated STEM-EELS and supported these observations with numerical simulations for both electron beam and light excitations.

**Figure 4:**
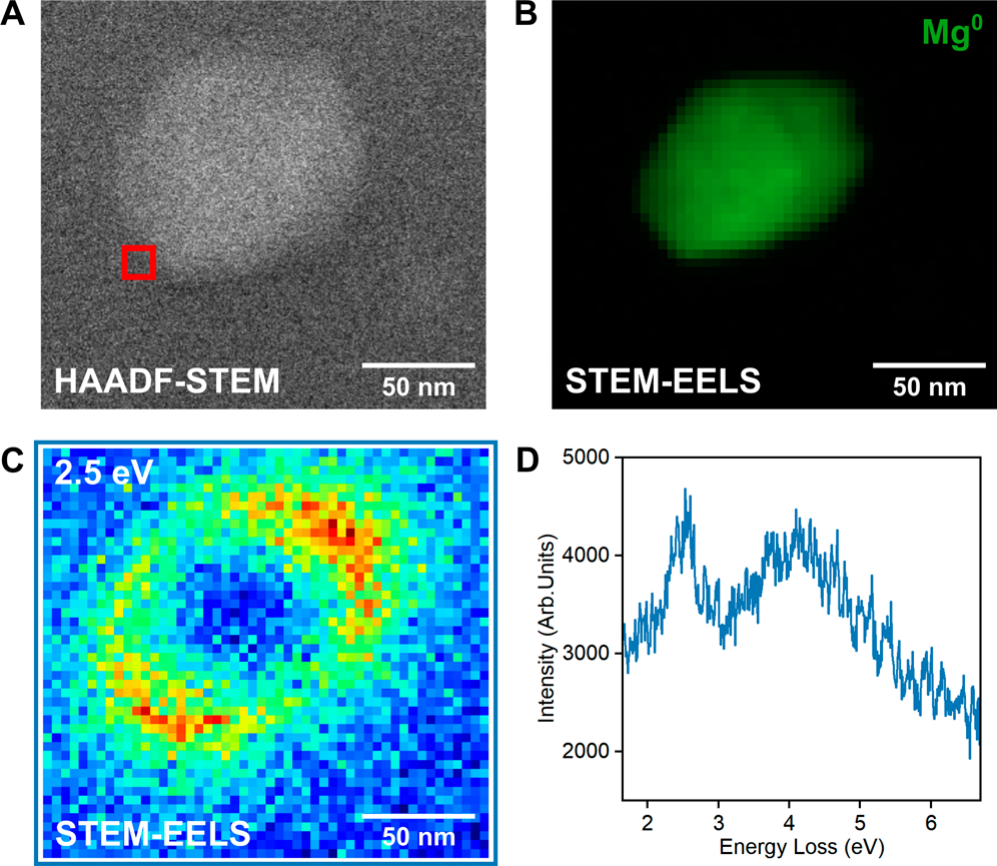
Optical properties of a single Mg faceted spheroid. (A) HAADF-STEM image, (B) STEM-EELS map of the metallic Mg bulk plasmon, (C) STEM-EELS loss probability map at the 2.5 eV dipolar LSPR obtained by integrating the loss signal from 2.45 to 2.55 eV, and (D) STEM-EELS point spectrum at the tip of the NP (binned over 3 × 3 pixels) from the red box in A.

LSPR modes from a single elongated Mg faceted spheroid were first probed with STEM-EELS. The most intense mode is the dipole resonance along the NP’s longitudinal axis near 2.5 eV, stretching beyond the oxide shell. Figure 4 shows the excitation probability from 2.45 to 2.55 eV, where this dominates. To extract spectral information and higher order modes, we also performed blind source separation with non-negative matrix factorization (NMF; Figure S15). A Lorentzian line shape fitted to the longitudinal dipole peak in the NMF spectral component revealed the peak energy to be 2.46 eV (504 nm). This resonance energy is comparable to the extinction peak observed in bulk UV–vis/NIR (Figure S2), with minor differences attributed to the different dielectric environments. Modes at higher energies were also present and extracted with NMF, with the expected shape-dependent energies and distributions (Figure S15).

**Figure 5:**
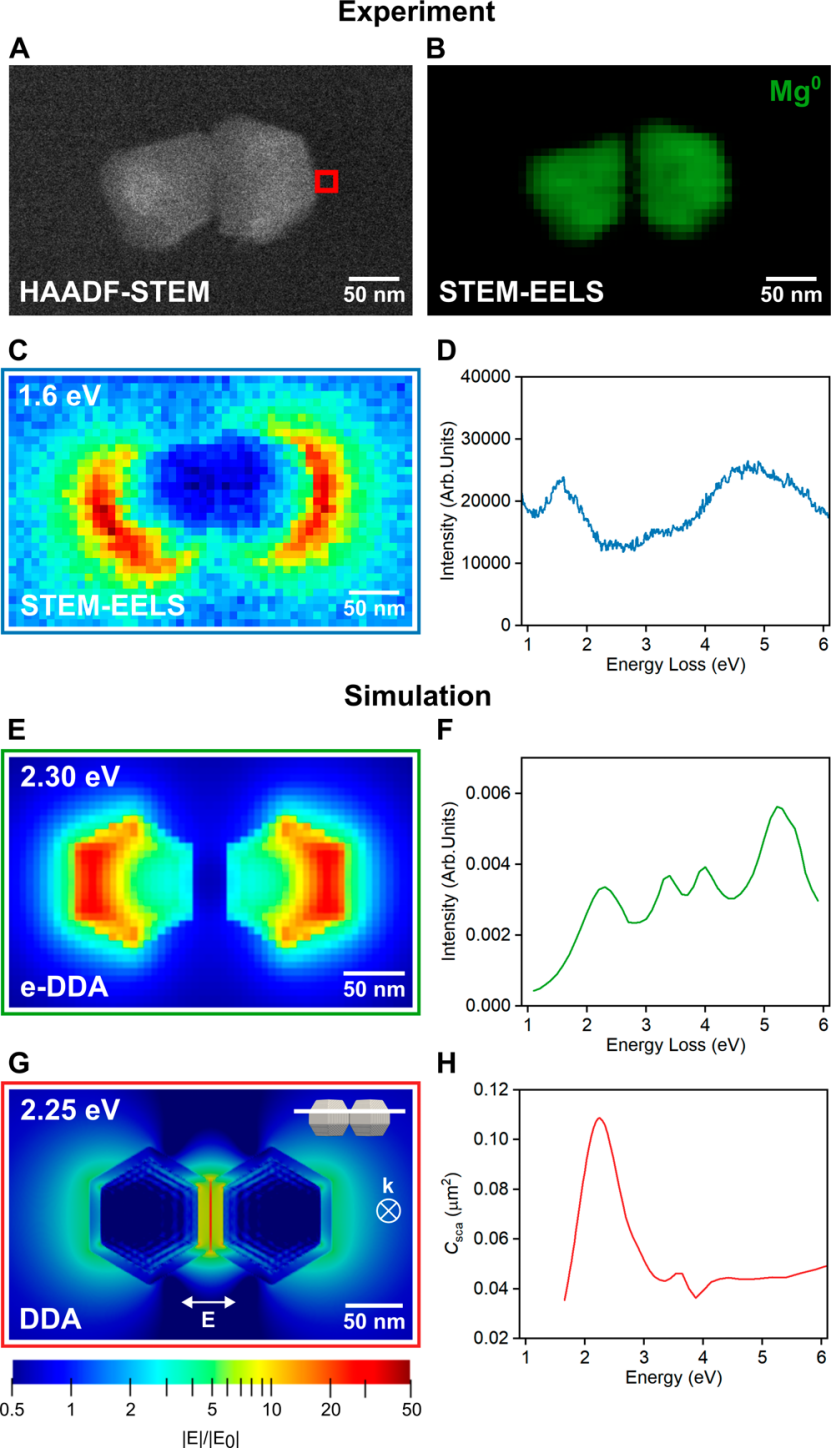
Optical properties of a Mg NP dimer. (A) HAADF-STEM image, (B) STEM-EELS map of the metallic Mg bulk plasmon, (C) STEM-EELS loss probability map at the 1.6 eV bonding dipolar LSPR obtained by integrating the loss signal from 1.55 to 1.65 eV, and (D) STEM-EELS point spectrum at the tip of the NP (binned over 2 × 2 pixels) from the red box in A. (E) e-DDA loss probability map and (G) DDA electric field distribution map at 2.25 eV of a dimer consisting of two Wulff-constructed Mg NPs with 10 nm of MgO layer, placed 2 nm apart along their facets and positioned on a 20 nm Si_3_N_4_ layer. (F) e-DDA point spectrum at the tip of the dimer (red box in A) and (H) DDA scattering cross section (*C*_sca_) of the dimer.

We then confirmed experimentally and numerically that dimers of plasmonic Mg NPs form prominent hot spots. In a dimer of any plasmonic NPs, modes oscillating along the long axis hybridize to bonding and antibonding modes.^82−85^ The former gives rise to an optically bright gap hot spot, while the antibonding resonance is optically dark.^85^ With an electron beam, it is possible to spatially map the excitation energy and localization, leading to mode identification, as shown above for an isolated NP, for both bright and dark modes. For the dimer in Figure 5A–D, we extracted a bonding mode energy of 1.64 eV (756 nm) with NMF. This mode shows strong electron excitation probability at the longitudinal tips of the dimer. As expected, there is no excitation in the interparticle gap because of the radial field symmetry of an electron that can interact with the antibonding mode instead. However, when excited with light, the bonding dipole is indeed the gap mode that leads to a strong enhancement, as simulated in Figure 5G. Higher order modes and antibonding gap modes are also observed at higher energies and reported in Figure S16.

Numerical results support the mode assignment and electron loss distribution. We performed simulations in the electron-driven discrete-dipole approximation (e-DDA)^82^ for a dimer consisting of Wulff construction-generated faceted spheroids (Figure S17).^86^ The total size of each NP, measured from tip to tip, is 120 nm, including a metallic Mg core and an outermost 10 nm MgO layer. The NPs were positioned facet-to-facet with a 2 nm interparticle gap and rested on a 20 nm Si_3_N_4_ layer, reproducing the experimental parameters as closely as possible. A simulated point spectrum for an electron beam trajectory just outside the dimer (Figure 5F) reveals features comparable to those obtained experimentally, with a distinct peak at low energy (2.30 eV) and multiple intense peaks at higher energies corresponding to the broad and intense experimental peak. The simulated electron loss probability map (Figure 5E) also agrees well with the experimental results (Figure 5C), depicting excitation of the bonding mode around the longitudinal tips of the dimer.

The thickness of the oxide layer has only minimal effects on the coupled dimer behavior. Further numerical simulations (Figures S18 and S19) were performed to compare the spectral response of dimers with increasing oxide layer thicknesses. The spectral features of the simulated STEM-EELS data (Figure S18) present only minor differences: the low energy peak red-shifts from 2.30 to 1.80 eV, while its relative intensity increases as the oxide shell is thinned from 10 nm to none.

DDA simulations performed on a Mg NP dimer excited by light reveal the formation of a hotspot and enable the determination of an oxide thickness-dependent field enhancement. We first observe that dimers, as discussed above, produce an electromagnetic hot spot when illuminated with light (Figure 5G). The highest scattering efficiency at the coupled dipolar peak occurs at 2.25 eV (Figure 5H) for a dimer with a 10 nm MgO shell. Numerical spectra of dimers with thinner oxide layers (Figure S19) reveal slight mode energy shifts and an increase in the scattering cross section for thinner oxide shells.

The calculated electromagnetic EF agrees well with the experimental results. We calculated the EF as |*E*|^4^/|*E*_0_|^4^ and used numerical inputs that match experimental conditions, including the surrounding IPA medium.^78,87^ We simulated excitation edge-to-edge in the monomer and along the interparticle axis for the dimer, then calculated their average electromagnetic EFs, *i.e.* average surface |*E*|^4^/|*E*_0_||^4^ (Table S4). The EF was calculated at 532 nm to match with the experimental excitation wavelength (Table 2), and at the corresponding maximum *Q*_sca_ (Table S4). Despite the infinity of possible particle and aggregate configurations, these simple models allow us to investigate the oxide layer effects on the EF, which are not addressable experimentally. Note that the alternative approximation using |*E*(ω)|^2^/|*E*_0_|^2^ × |*E*(ω′)|^2^/|*E*_0_|^2^, where ω and ω′ are maximum *Q*_sca_ and stoke-shifted frequencies,^8,88^ gives EFs comparable to those of |*E*|^4^/|*E*_0_|^4^ (Table S4).

The numerical EFs calculated at 532 nm for the monomer and the dimer are 45 and 119, respectively, in the presence of a 10 nm oxide layer and 93 and 33533, respectively, in its absence (Table 2 and Figure S20). The experimental EFs are of the same order of magnitude as the EF values calculated for 5 and 10 nm oxide layers. This is consitent with the oxide layer thickness obtained from STEM-EDS (Figure 1E).

**Table 2:** Calculated Average Electromagnetic EFs of a Mg NP Monomer and Dimer at 532 nm with Varying Oxide Layer Thickness

Oxide layer thickness (nm)	Monomer EF	Dimer EF
0	93	33533
5	73	341
10	45	119

### Binding, SERS Spectra, and EF of 4-MBA and 4-NBT on Mg–Pd NPs

**Figure 6:**
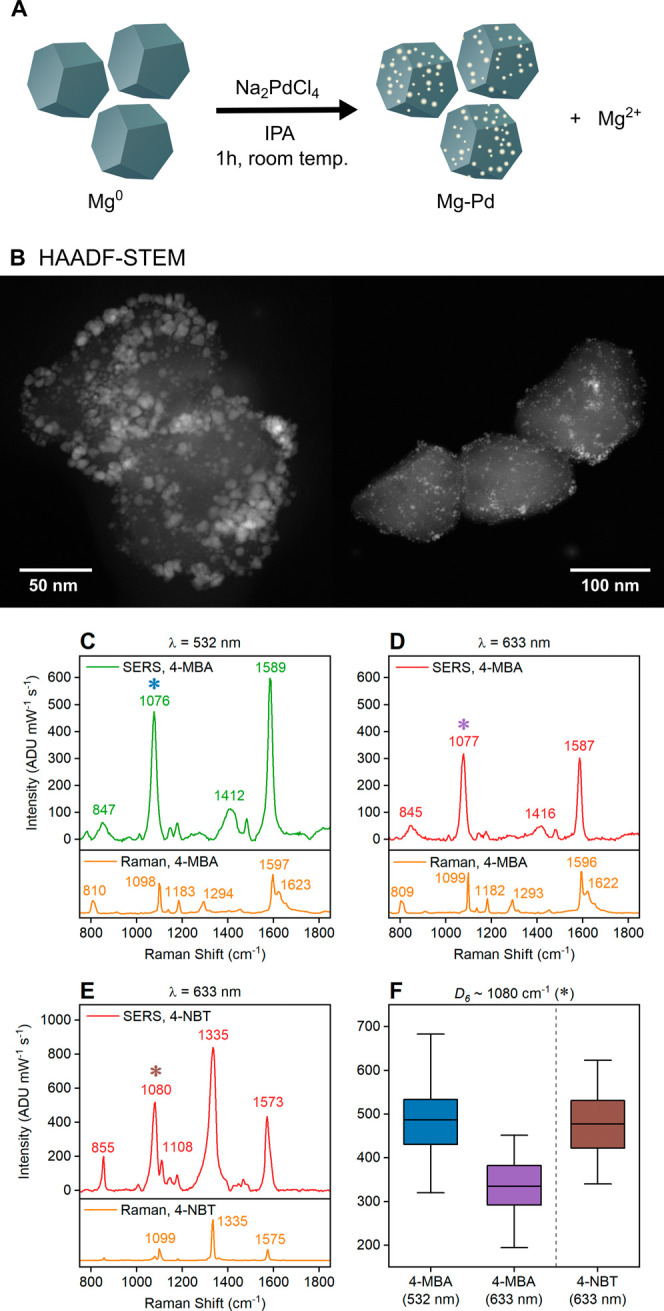
Mg–Pd NPs obtained by partial galvanic replacement and their SERS spectra. (A) Schematic of the synthetic approach. (B) HAADF-STEM images of Mg–Pd NPs. SERS spectra using (C) 4-MBA at 532 nm, (D) 4-MBA at 633 nm, and (E) 4-NBT at 633 nm. Normal Raman spectra of analytes in solid form at the same laser wavelength are shown under each SERS spectrum as a reference. (F) Variation of intensity of the peak ∼1080 cm^–1^ (*D*_6_ mode, labeled with a colored asterisk in A–C), across 100 randomly selected regions. Full raw spectra without background subtraction are reported in Figures S24 and S8.

Mg NPs decorated with 3.3 mol % Pd (Mg–Pd NPs) enhance the electric near-field for SERS. Mg–Pd NPs were synthesized by partial galvanic replacement of colloidal Mg NPs by Na_2_PdCl_4_ (Figure 6A), as previously reported.^49^ Successful decoration of Mg NPs with Pd is confirmed by STEM-EDS (Figure S21) and HAADF-STEM images (Figures 6B and S22). The Pd content was measured by ICP-OES to be 3.3 mol % (Table S1), and no change to the LSPR peak in the UV–vis/NIR extinction spectrum was observed (Figure S23), confirming the retention of the majority of the plasmonic, metallic Mg core.

4-MBA and 4-NBT bind to Mg–Pd NPs during incubation and remain on the surfaces of NPs after cleaning. Indeed, thiols have been demonstrated to bind to Pd surfaces,^89−91^ and thus with Mg–Pd, 4-MBA and 4-NBT are bound both to Pd sites and to MgO surfaces.

The SERS measurements of Mg–Pd NPs were first performed on dry NPs deposited on membrane filters (Figure S5), as was done with Mg NPs. The SERS spectra of 4-MBA and 4-NBT adsorbed on dry Mg–Pd NPs (Figure 6C–F) differ from those of Mg NPs. First, the background in the Mg–Pd SERS spectra of 4-NBT is lower than that with the Mg NPs (Figure S24), likely due to fluorescence quenching from the metallic Pd NPs. Second, the relative peak intensities of the *D*_6_ (1080 cm^–1^) and *D*_3_ (1593 cm^–1^) modes to all other peaks are higher on Mg–Pd than those on Mg NPs. The N–O stretching mode of the 4-NBT anion at 1295 cm^–1^ is not observed on Mg–Pd NPs, though it may be obscured by the peak at 1335 cm^–1^, which is broader on Mg–Pd compared to Mg NPs. No change over time in the spectra of 4-MBA at 532 and 633 nm and 4-NBT at 633 nm has been observed.

The change to the relative peak intensities suggests a different binding geometry on Pd sites compared to MgO. The geometry can be deduced from the SERS spectra of 4-MBA. The absence of the C=O vibration at 1710 cm^–1^ indicates that 4-MBA is deprotonated^45,48,60^ and hence that the lower relative intensities of the COO^–^ peaks are not due to the presence of COOH. Similarly, the absence of S–H modes at 2580 and 915 cm^–1^^45,48^ and the presence of the carboxylate anion peak near 1416 cm^–1^ are consistent with binding through S and not COO^–^.^45,64^ So far, these geometry indicators match those of 4-MBA on the Mg NPs. However, in Mg–Pd NPs, the out-of-plane ring vibration mode (estimated to be *D*_17_) and the *D*_4_ mode (containing C–H in-plane bending) are present near 718 (Figure S24) and 1482 cm^–1^ (Figure 6C,D), respectively, indicating analyte molecules positioned flat or at an angle to the Pd surface.^46,48,60^ The *D*_1_ and *D*_2_ modes (containing aromatic C–H stretching) at 3063 cm^–1^ (Figure S24) stem from a perpendicularly oriented 4-MBA and are still present in the 532 nm Mg–Pd SERS spectrum, confirming that analytes bound to MgO sites have the same perpendicular binding geometry as they do on Mg NPs.^64,66^

**Figure 7:**
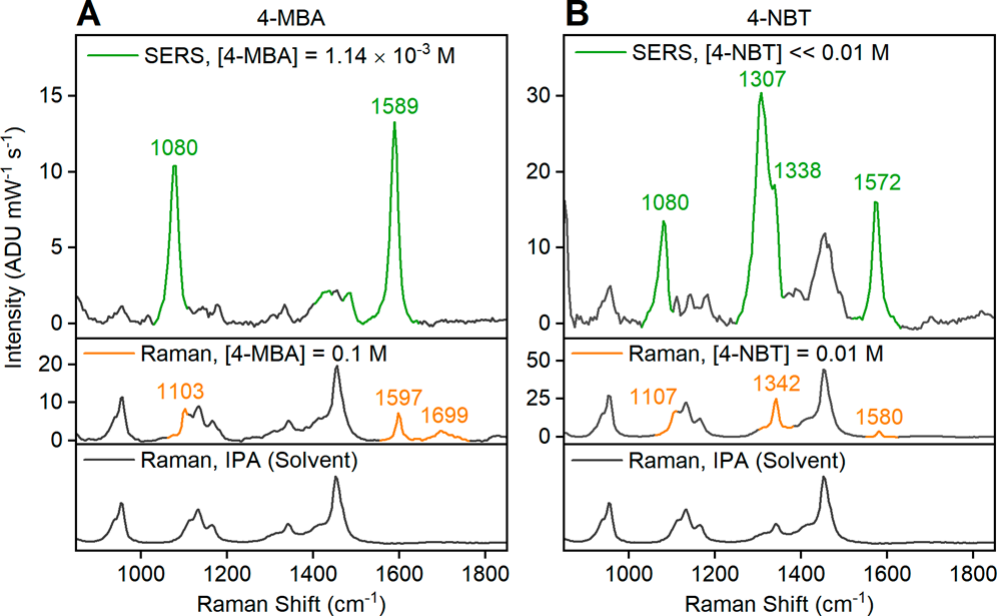
SERS spectra of analyte-incubated colloidal Mg–Pd NPs dispersed in IPA. (A) 4-MBA and (B) 4-NBT were used at 532 nm. The normal Raman spectra of 0.1 M 4-MBA and 0.01 M 4-NBT solution in IPA, as well as the spectra of IPA are shown as a reference, under the SERS spectra. The spectral features of IPA are plotted in black, while the peaks from analytes are highlighted in color. The 4-MBA concentration in SERS were quantified with ICP-OES (Table S1). The 4-NBT concentration in SERS are estimated from the incubation solution concentration and subsequent cleaning steps. Equivalent SERS data at 633 nm and full raw spectra without background subtraction are reported in Figures S25–S27 and S11.

The SERS peaks in the colloids (Figures 7, S25–S27) match those from dry Mg–Pd NPs. The 4-MBA SERS peaks in colloidal Mg–Pd NPs are identical to those on dry Mg–Pd NPs at both wavelengths, indicating an equivalent orientation of the molecule on the surface. Similarly, the 4-NBT signals are also equivalent at 633 nm, although the N–O stretching mode (1338 cm^–1^) appears broadened at lower energy in colloids, implying the presence of a N–O stretching mode from 4-NBT^–^. At 532 nm excitation, the N–O stretching mode of 4-NBT^–^ at 1307 cm^–1^ is pronounced and has a higher relative intensity compared to that of the neutral species.

The EF of Mg–Pd colloidal NPs based on the monolayer surface coverage approximation is calculated to be ∼10^3^, an order of magnitude higher than that of Mg NPs (Table 1). The calculation employs Mg and Pd concentrations from ICP-OES (Table S1), and 12.007 g cm^–3^ for the density of Pd,^92^ in addition to the parameters described previously. *N*_Surf_ is calculated from the total surface area for Mg–Pd NPs, assuming 121 nm spheres of Mg containing the amount of Pd determined by ICP-OES (Table S1), such that the surface area is similar to that of Mg spheres. An alternative approach to calculating the surface area, using Pd spheres on Mg spheres, led to substantially similar values (SI and Table S5). As with Mg NPs, the EFs of Mg–Pd NPs are higher at 633 nm excitation than at 532 nm. A calculation using the ICP-OES-determined S content led to an EF in the order of 10^2^ (Table S6), a value larger than that for Mg NPs, yet smaller than the surface coverage estimate due to the overestimation of *N*_Vol_, as discussed previously.

The higher EF in Mg–Pd NPs compared to Mg NPs is a result of the different orientation taken by 4-MBA and 4-NBT. As described earlier, some molecules are oriented at an angle on the surface of Mg–Pd NPs, while they were perpendicular to the surface of Mg NPs. As a result, *D*_6_ (1080 cm^–1^) and *D*_3_ (near 1580 cm^–1^) modes are enhanced further in Mg–Pd NPs.^46^ Because the *D*_3_ mode was the only peak available without an overlap with the signal from IPA, it was used for the calculation of the EF. Without the additional enhancement induced from the orientation of the molecule, the magnitude of electromagnetic enhancement from plasmonic Mg–Pd NPs is likely similar to that of Mg NPs.

### Coupling Reaction of 4-NBT to DMAB on Mg and Mg–Pd NPs

**Figure 8:**
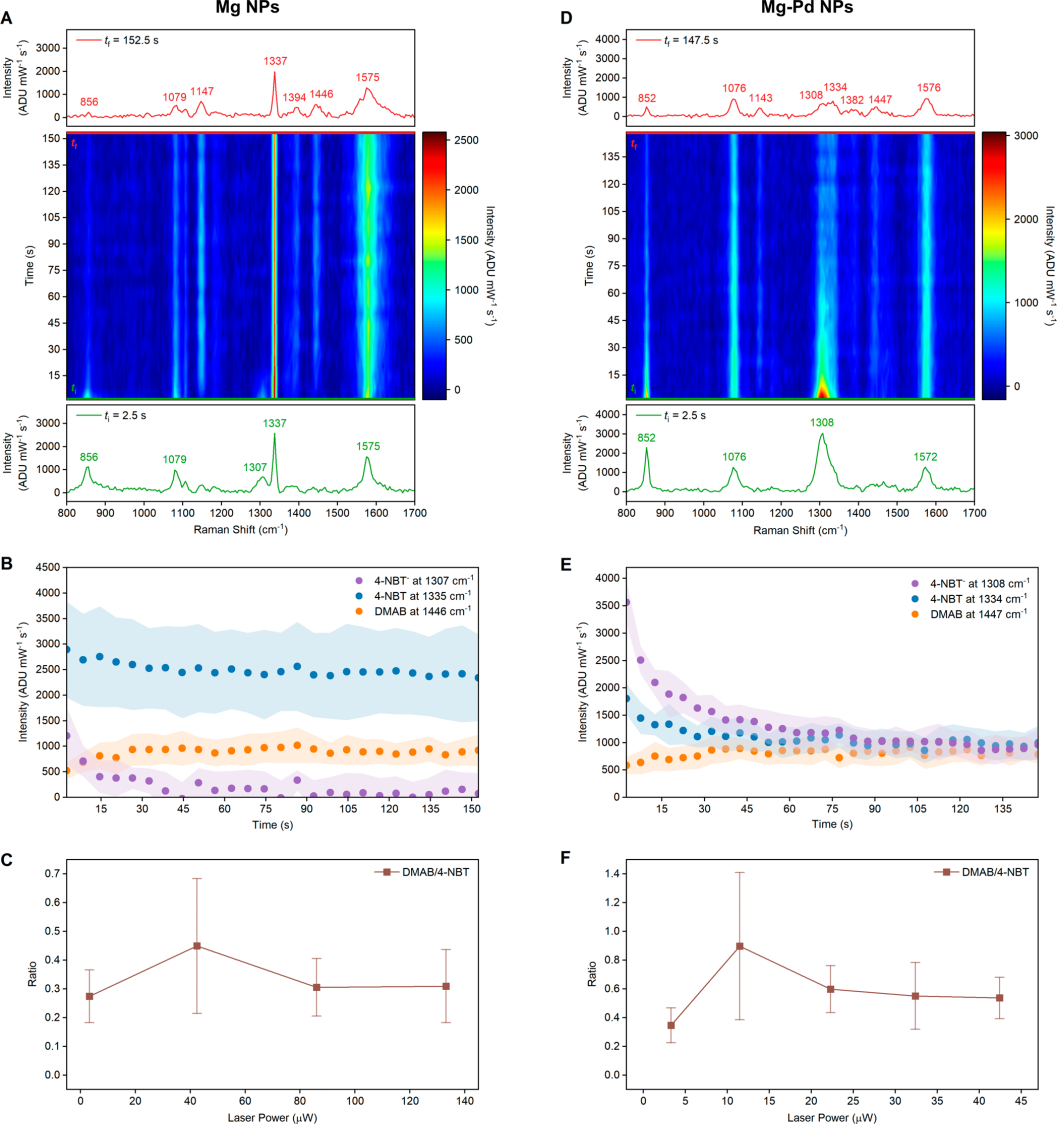
SERS dynamics during the reductive coupling reaction of 4-NBT to DMAB. Spectra were acquired on dry (A–C) Mg and (D–F) Mg–Pd NPs at 532 nm using a 5 s acquisition time with time, *t*, set as the midpoint of each acquisition. The evolution map of SERS spectra over time at (A) 42.4 and (D) 11.5 μW laser power. The spectra at *t*_i_*=* 2.5 s and *t*_f_ = 152.5 (*t*_f_ = 147.5 for Mg–Pd in D) are shown in green and red, respectively. The full raw spectra without background subtraction at *t*_i_ and *t*_f_ are presented in Figures S28 and S31. (B, E) The change in peak intensities over time of the N–O stretching modes and the −N=N– stretching mode from 4-NBT^–^, 4-NBT, and DMAB, respectively. The data points represent the average peak intensities and the colored backgrounds represent their standard deviation, *N* = 20. (C, F) Ratios of DMAB (1446 cm^–1^ for Mg and 1447 cm^–1^ for Mg–Pd) to 4-NBT (1337 cm^–1^ for Mg and 1334 cm^–1^ for Mg–Pd) calculated at *t*_f_ at varying laser power; error bars showing the standard deviation.

The SERS spectra of 4-NBT on dry Mg NPs at 532 nm (Figures 8A, S28, and S29) indicate the formation of DMAB, a product of the reduction of 4-NBT molecules, through a widely reported plasmon-driven reductive coupling reaction.^93^ Time-resolved spectra show that the intensity of the N–O stretching mode from 4-NBT (1337 cm^–1^) decreases while peaks corresponding to DMAB at 1147, 1394, and 1446 cm^–1^ gradually evolve. The peak at 1147 cm^–1^ can be assigned to the C–N stretching mode, while the peaks at 1394 and 1446 cm^–1^ are −N=N– stretching modes of DMAB.^32,94^

The reaction also produces 4-NBT^–^ species, evidenced by the presence of the N–O stretching mode at 1307 cm^–1^. The intensity of this peak decreases rapidly in the early stages of the reaction and eventually vanishes. The disappearance of the 4-NBT^–^ species could indicate its conversion to DMAB and/or desorption in the form of 4-NBT.

Not all 4-NBT molecules are converted into DMAB. Across the four laser powers used (3.3, 42.4, 86.0, and 133.2 μW at the sample), none led to the complete conversion of 4-NBT to DMAB, as evidenced by the presence of the N–O stretching mode of 4-NBT at 1337 cm^–1^ even after the DMAB signal plateaus (Figures 8B and S30). The reactions appear to be irreversible: the spectra did not revert to their initial form nor undergo further changes even after prolonged periods without laser irradiation.

The catalytic performance can be assessed based on the ratio of the DMAB to the 4-NBT peaks after the DMAB signal reaches a plateau. Here, we use the ratio between the peaks at 1446 (DMAB) and 1337 cm^–1^ (4-NBT) after 152.5 s of reaction time. The peak ratio is known to increase with laser power as a result of the increased conversion,^32,75^ and more specifically, in areas with higher electromagnetic field.^31^ Here, the ratio varied slightly from region to region owing to enhancement differences, and therefore, the data in Figure 8C report the average of 20 regions. The ratio of the DMAB to the 4-NBT peaks is 0.27, 0.45, 0.31, and 0.31, at 3.3, 42.4, 86.0, and 133.2 μW, respectively (Figure 8C).

The resulting DMAB to 4-NBT peak ratio is lower than the 0.56 reported for Ag NPs,^31^ as expected due to the lower field enhancement of MgO-covered Mg. The decrease in the ratio with higher laser power is attributed to the higher rate of desorption, indicated by the decline of both 4-NBT and DMAB peak intensities (Figure S30). The slope of the decline increases with laser power and is steepest at 133.2 μW. This desorption dependence on laser power has previously been reported for 4-NBT on Au NPs,^68^ with a presumed involvement of plasmonic effects beyond photothermal heating.^72^

The plasmon-driven reductive coupling of 4-NBT is believed to proceed via transferring the hot carriers, produced from plasmonic NPs, to the molecules on the surface.^72,95^ Although Golubev et al. highlighted the contribution from thermal effects,^96^ and Mg NPs can produce heat output comparable to Au NPs,^97^ Keller and Frontiera suggested that heat is not the dominant mechanism.^98^ Here, the reaction proceeding at 532 nm, where Mg’s photothermal efficiency is lower,^97^ but not at 633 nm, supports the hot carrier-mediated mechanism despite the oxide layer. Medeghini et al. recently showed that a small amount of hot carriers from Au nanorods can pass through a mesoporous silica layer of similar thicknesses to the MgO layer in Mg NPs (∼10 nm),^99^ supporting this observation. An alternative mechanism could involve the O_2_^–^ radicals formed in the oxygen vacancies of MgO.^100^ Indeed, the hot carrier origin and facilitating role of O_2_^–^ radicals in 4-NBT coupling has been proposed by Zhang et al.^101^ In this study with Mg NPs, we are unable to unequivocally discern a dominant mechanism behind the reductive coupling of 4-NBT.

The coupling reaction of 4-NBT on Mg–Pd NPs (Figures 8D, S31, and S32) proceeds with several differences compared to Mg NPs. First, the rate of 4-NBT to DMAB transformation is higher on Mg–Pd NPs than on Mg NPs and reaches a plateau quicker at equal laser power, as expected due to the presence of catalytically active Pd (Figures 8E and S33). Consequently, we chose lower laser powers (3.3, 11.5, 22.3, 32.4, and 42.4 μW) for the time-resolved SERS spectra of Mg–Pd NPs (Figure 8E). Second, more 4-NBT^–^ is produced on Mg–Pd NPs at the initial stage of the reaction, as revealed by the early appearance of an intense N–O stretching mode at 1308 cm^–1^. Unlike with Mg NPs, the anion peak is initially more intense compared to the same mode in 4-NBT (1334 cm^–1^), indicating the increased proportion of 4-NBT^–^; it eventually falls below that of the 4-NBT. Since the 4-NBT^–^ peak is not observed at 633 nm, the dominance of the peak at 532 nm suggests that the 4-NBT^–^ formation is driven by electromagnetic field irradiation. Finally, the presence of Pd on Mg NPs increases the ratio of the DMAB (1447 cm^–1^) to the 4-NBT peaks (1334 cm^–1^) after 147.5 s of reaction time (Figure 8F). The ratio is highest under 11.5 μW laser power, at 0.90, which is double the maximum from Mg NPs. As seen with Mg NPs, the ratio of the DMAB to the 4-NBT peaks decreased with a higher laser power likely due to desorption processes taking place at higher powers. Still, when comparing at equal laser power, the ratio at 42.4 μW with Mg–Pd NPs is 0.54, i.e., 20% higher than the ratio obtained on Mg NPs at this power. With the LSPR of Mg coupling to Pd, hot carriers could be generated in the Pd and transferred to 4-NBT, similarly to what was observed for Pd-decorated, oxide-coated Al NPs.^33^ Note that the differences in the ratios between Mg–Pd and Mg NPs can be overestimated since 4-NBT^–^, produced in larger proportion on Mg–Pd, is not included in the calculations.

## Conclusion

We demonstrated the application of Mg and Mg–Pd faceted spheroids for SERS and SERS-trackable plasmon-driven catalysis. The SERS EF measured using 4-MBA and 4-NBT analytes at 532 and 633 nm was on the order of 10^2^ and 10^3^ for Mg and Mg–Pd NPs, respectively. LSPR modes in Mg NPs and dimers were experimentally mapped and indicated that dimers formed coupled modes, as confirmed by numerical simulations with electron beam and light excitations. Simulations also provided a calculated value for the EF of Mg NP dimers, which matches the experimental results.

By analyzing the SERS spectra of 4-MBA, we determined that the analyte was bound to the surface of NPs through S, with a perpendicular orientation on MgO surfaces and a tilted or flat orientation on Pd surfaces. While the decarboxylation of 4-MBA was not observed, 4-NBT was converted to DMAB on the surface of dry Mg and Mg–Pd NPs under 532 nm excitation through a plasmon-driven reductive coupling reaction. The final SERS peak ratio of DMAB to 4-NBT was higher for Mg–Pd NPs than Mg NPs, and the former also displayed a higher rate of reaction. Whereas in Mg–Pd, the reaction can be mediated by hot carriers generated in Pd due to the near field effects in plasmonic Mg, the mechanism of the coupling on Mg NPs is not fully understood and requires further investigation.

The results presented here further validate the applicability of Mg NPs as plasmonic material. Mg’s capability to form SERS substrates provides potential in sensing applications. The demonstration of molecular binding through S suggests that the rich library of approaches relying on Au–S affinity could be applicable to Mg NPs. Further, the Mg NPs’ ability to drive light-induced reactions on their surface, confirmed by SERS, solidifies Mg’s attractiveness as an earth abundant platform for plasmon-enhanced catalysis.

## Methods

### Materials

Li pellets (99%), naphthalene (99%), 1.0 M di-*n*-butylmagnesium (MgBu_2_) in heptane, poly(vinylpyrrolidone) (PVP, average molecular weight = 10,000), 4-mercaptobenzoic acid (99%), 4-nitrobenzenethiol (80%), sodium tetrachloropalladate (Na_2_PdCl_4_, 99.99%), gold(III) chloride trihydrate (99.9%), ethylenediamine tetraacetic acid (EDTA, 99.995%), nitric acid (70%), anhydrous tetrahydrofuran (THF), anhydrous isopropanol (IPA), and Millipore Express PLUS 0.22 μm Poly(ether sulfone) (PES) membrane filters (hydrophilic, nonsterile, diameter = 47 mm) were purchased from Sigma-Aldrich and used as supplied. Hydrochloric acid (37%) was purchased from VWR Chemicals. Sodium citrate dihydrate and citric acid were purchased from Fisher BioReagents. The ICP standards (Mg, S, Pd, and Au) were purchased from Sigma-Aldrich or Acros. All glassware were washed with nitric acid and flame-dried under vacuum before use. Quartz cuvettes (3500 μL, enhanced chemical resistance) equipped with septum screw caps were purchased from Thorlabs. The cuvettes were immersed in 2 M nitric acid overnight or in aqua regia (**WARNING! Aqua regia is extremely corrosive and highly oxidizing. Handle with extreme caution and never add organics to aqua regia.**) for 10 min if Pd containing sample was used in previous use and rinsed with distilled water and ethanol before use. Optical quality borosilicate coverslips (thickness #1) were purchased from Agar Scientific and used as supplied.

### Synthesis of NPs

Mg faceted spheroids were synthesized using the previously reported one-pot seed-mediated growth method.^40^ Briefly, di-*n*-butylmagnesium (MgBu_2_) in heptane (1.75 mL, 1.0 M) was injected quickly into freshly prepared Li_2_Napht solution containing poly(vinylpyrrolidone) (20 mg) in a Schlenk flask under an Ar atmosphere at room temperature and sonication (**WARNING! MgBu_2_is pyrophoric and should be handled under inert conditions.**). Naphthalene in THF (2 mL, 1.0 M) was added to the reaction mixture after 5 min reaction time, converting all unreacted Li_2_Napht to LiNapht. The resulting mixture was left to react for further 60 min before being quenched by the injection of IPA (2 mL). The solid gray product was recovered by centrifugation (10,000 rcf) and residual byproducts were removed by rounds of centrifugation (10,000 rcf) and redispersion steps in THF twice, IPA once, THF once, and IPA twice, in the listed order under inert conditions. The product was redispersed in IPA (15 mL).

Mg–Pd NPs were prepared by partial galvanic replacement of colloidal Mg faceted spheroids using the previously reported procedure.^49^ In brief, colloidal Mg NPs (1 mL) were diluted with IPA (2 mL) before adding a solution of sodium tetrachloropalladate (Na_2_PdCl_4_) in IPA (3 mL). The stoichiometric amount of Na_2_PdCl_4_ was calculated based on the Mg content of as-synthesized Mg NPs obtained from ICP-OES. The resulting mixture was left to react for 1 h in a sealed vial under stirring. The solid gray product was recovered by centrifugation and residual byproducts were removed by rounds of centrifugation and redispersion steps in IPA three times. The product was redispersed in IPA (8 mL).

Au NPs (46 nm, citrate capped) were synthesized using a seeded-growth method. Au NP seeds (12 nm, citrate capped) were synthesized using a modified Turkevich methodology described by Schulz et al.^102^ In brief, a solution (80 mL) containing citrate buffer (3:1 trisodium citrate/citric acid, 2.75 mM) and ethylenediamine tetraacetic acid (EDTA, 0.02 mM) was heated to boil under vigorous stirring for 10 min before adding an aqueous solution of gold chloride (0.8125 mM, 20 mL). The resulting reaction was left to boil for further 20 min under stirring, during which the reaction mixture turned red, producing Au NP seeds. The Au NP seeds were grown using the growth method reported previously,^103^ involving successive additions of gold chloride and citrate. Briefly, trisodium citrate (34 mM, 2 mL) was added to distilled water (82.5 mL) and heated to boil. As-synthesized Au NP seeds (2 mL) were added to the boiling mixture, followed by the addition of gold chloride (6.8 mM, 1.7 mL) after 1 min. The reaction mixture was heated to reflux for 45 min. Further successive additions of trisodium citrate (34 mM, 2 mL) and gold chloride (6.8 mM, 1.7 mL) were performed 5 times, with the mixture remaining under reflux for 45 min between growth steps. The size of the NPs was determined using UV–vis/NIR spectroscopy with the method proposed by Haiss et al. and confirmed with SEM.^104^ The resulting colloidal Au NPs (40 mL) were centrifuged (5000 rcf) and redispersed in IPA (10 mL) before use.

### SERS and Raman Measurements

As-synthesized Mg NPs (5 mL) were mixed with a solution of Raman reporter molecules (4-MBA or 4-NBT, 0.01 M, 5 mL) in IPA under inert atmosphere and incubated overnight. The excess Raman reporter molecules were removed by three rounds of centrifugation (10,000 rcf) and redispersion steps using IPA, and the resulting NPs were redispersed in IPA (2 mL), all under inert conditions. The above procedure was repeated with Mg–Pd NPs (4 mL) and Au NPs (10 mL) using equal volumes of Raman reporter molecules solutions (0.01 M) to NPs, but unlike with Mg NPs, Mg–Pd NPs were redispersed in 1.6 mL of IPA, while Au NPs were redispersed in 2 mL of IPA. The resulting colloidal NPs (∼1.4 mL) were transferred to a cuvette under inert atmosphere and sealed with a septum screw cap for colloidal SERS measurements. For SERS measurements of dry NPs, colloidal NPs (100 μL) were deposited by a continuous feeding of NPs on PES membrane filters under vacuum.

SERS and Raman spectroscopy were performed using a HORIBA Jobin Yvon LabRam 300 Raman system equipped with a CW 532 nm Nd:YAG laser (up to 500 mW power), a 633 nm HeNe laser (20 mW power), O.D. filters ranging from 0.01 to 100%, Olympus BXFM-ILHS microscope with motorized *z*-axis of freedom, a motorized *x*,*y*-adjustable stage, HORIBA Syncerity detector, and LabSpec 6 Spectroscopy Suite software. An Olympus LMPlanFl 50×/0.50 objective and a 600 g/mm grating were used for all measurements reported here. The beam diameter at sample was 2.5 μm. The laser power at sample position was measured using a Thorlabs slim Si sensor (400–1100 nm, 500 pW–500 mW) connected to a Thorlabs PM100D digital console. All data were converted to analogue to digital converter units (ADU) before analysis by the division of laser power at sample position and integration time. Data analysis was performed using Origin Pro.

SERS on 4-MBA-bound dried NPs on membrane filters was conducted using 86.0 μW (532 nm) and 54.0 μW (633 nm) laser power at sample position. 100 regions were randomly selected across the layer of each sample and a SERS spectrum at each region was collected by averaging over 3 consecutive acquisitions, each with 30 s integration time. For every sample, spectra from 100 regions were averaged before a background was fitted by Spline polynomial interpolation and then subtracted. The peak at 1080 cm^–1^ was fitted using the unaveraged spectra from 100 regions individually, by subtracting a linear background between regions before and after each peak and finding the maximum intensity within the region, to obtain intensity distribution.

SERS on 4-MBA-bound dried single NPs and single aggregates drop-cast on coverslips was conducted using 86.0 μW (532 nm) laser power at sample position. The SERS spectrum at each region of interest was acquired by averaging over 10 consecutive acquisitions, each with 60 s integration time.

SERS on colloidal NPs was performed using 3.84 mW (532 nm) and 2.0 mW (633 nm) laser power at sample position. The cuvettes containing the samples were positioned under the microscope and the beam was focused on the colloidal region closest to the cuvette surface. For each SERS measurement, a normal Raman spectrum was acquired using solutions of Raman reporter molecules (0.1 M solution of 4-MBA in IPA and 0.01 M solution of 4-NBT in IPA) to allow the evaluation of EF using peak intensity. The focus position was kept constant between SERS and normal Raman acquisitions of same Raman reporter molecules. SERS and normal Raman spectra were acquired 10 times for each sample, with every measurement being averaged over 3 consecutive acquisitions each with 30 s integration time. Samples were mixed by shaking the cuvettes in between measurements to ensure an even distribution of NPs. For every sample, spectra from 10 measurements were averaged before background was fitted by Spline polynomial interpolation and then subtracted.

SERS-based monitoring of the plasmon-driven coupling of 4-NBT to DMAB was carried out using the 532 nm laser at stated laser powers. Unless otherwise stated, time-series SERS spectra were acquired at 20 individually selected regions for each sample, using 5 s acquisition time for a total of 150 s at each region. Acquisition for each region began at the time the laser was turned on. The half point time in each acquisition from turning on the laser was used as time stamp. For every time stamp from the same sample, spectra from 20 regions were averaged before background was fitted individually by Spline polynomial interpolation and then subtracted, unless specified otherwise. The peaks (1307 cm^–1^ for 4-NBT^–^, 1338 cm^–1^ for 4-NBT, and 1447 cm^–1^ for DMAB) were fitted using the unaveraged spectra individually by subtracting a linear background between regions before and after each peak and finding the maximum intensity within ±5 cm^–1^ peak window. The peak intensities were then averaged at every time stamp for the same sample. The ratio of DMAB/4-NBT was calculated by dividing the DMAB intensity by 4-NBT intensity from each unaveraged spectrum and then averaging the ratio at every time stamp for the same sample.

### Characterization of NPs

UV–vis/NIR spectroscopy was performed using Thermo Fisher Evolution 220 UV–visible spectrophotometer with the sample in a PMMA semimicro cuvette at room temperature.

SEM imaging of as-synthesized Mg NPs drop-cast on Si wafers and of 4-MBA-bound Mg NPs drop-cast on borosilicate coverslips was performed on FEI Nova NanoSEM operated at 5 kV and equipped with an Everhart-Thornley detector for secondary electron imaging. The latter were carbon coated prior to imaging. Mg NPs on membrane filters were cut-out, attached to glass coverslips, and carbon coated for SEM imaging, which was performed on FEI Quanta-650F Field Emission Gun SEM operated at 5 kV and equipped with an Everhart-Thornley detector for secondary electron imaging.

TEM imaging and STEM-EDS line scans of Mg–Pd NPs drop-cast on a 10 nm thick Si_3_N_4_ membrane was performed on FEI Tecnai Osiris operated at 200 kV and equipped with a Gatan UltraScan1000XP (2048 by 2048 pixel) camera and an FEI Super-X quadruple EDS detector. STEM-EDS line scans were processed using an open-source software, Hyperspy.^105^ For Mg Kα (1.25 keV) and Pd Lα (2.84 keV) lines, a linear background was fitted between the regions below and above the peaks, and lines were integrated above the background to obtain elemental distribution. The integration was set to the extended energy resolution of Mn Kα from the detector.

HAADF-STEM, STEM-EDS, and STEM-EELS of Mg NPs with Raman reporter molecules were acquired on Thermo Fisher Spectra 300 TEM equipped with a high energy resolution extreme field emission gun monochromator (X-FEG Mono), a Panther segmented STEM detector, an FEI Super-X quadruple EDS detector, and a Gatan Continuum EELS detector. The monochromator was tuned as required by the desired energy resolution. Mg NPs were cleaned three times after incubating with Raman reporter molecules, 4-MBA or 4-NBT, before being drop-cast on a 10 nm thick Si_3_N_4_ membrane for STEM. STEM-EDS and STEM-EELS data were processed using an open-source software, Hyperspy.^105^ For STEM-EDS, Kα lines of Mg (1.25 keV), O (0.52 keV), S (2.31 keV), and C (0.28 keV) were integrated following the procedure described above. With STEM-EELS, the ZLP was used to align the energy axis with subpixel accuracy, and spikes were removed by linear interpolation. The map of Mg bulk plasmon was obtained by summing the spectra between 9.0 and 11.0 eV. Other energy slices were obtained by summing the spectra over the specified energy range. For NMF analysis, the spectra were cropped to the range of 0.3 to 8.0 eV and minimum intensity was shifted to 0 before extracting modes. The optimum number of NMF components was determined by trial-and-error, with the largest value which did not cause the duplicate factorization of identical components being selected. A Lorentzian line shape was fitted to peaks in NMF spectral factors to determine the energy of the peak.

ICP-OES analysis was performed on Thermo Fisher Scientific iCAP 7400 Duo ICP-OES Analyzer. Mg NPs were digested in an aqueous matrix with dilute nitric acid, while Mg–Pd NPs were digested in an aqueous matrix with aqua regia (**WARNING! Aqua regia is extremely corrosive and highly oxidizing. Handle with extreme caution and never add organics to aqua regia.**). Samples were diluted to ∼1 ppm (mg L^–1^) for analysis.

Single particle/aggregate optical dark field spectroscopy was performed on Mg NPs drop-cast on borosilicate coverslips in air. The scattering spectra were obtained using an optical set up equipped with a Nikon Eclipse Ti inverted microscope, a Physik Intrumente P-545.3C7 piezoelectric stage, a halogen lamp, a dark field condenser (numerical aperture, NA of 0.85–0.95), a 100× oil immersion objective (Variable NA set to <0.8), a Princeton Instruments IsoPlane 320 spectrometer with a 50 g/mm grating, and a PIXIS 256 detector. The exposure time was set to 1 s with 4 frames accumulated per position.

### Numerical Methods

Optical scattering spectra were obtained numerically in the discrete dipole approximation (DDA) using DDSCAT.^106^ EELS calculations were performed using e-DDA,^15,82^ a version of DDSCAT modified to replace the plane wave stimulation with a swift electron beam. Input NP shapes for DDA and e-DDA were obtained using the HCP Wulff construction function of Crystal Creator, a freely available crystal shape modeling tool.^41,86^ The faceted spheroids were modeled using surface energy values from Lautar et al., yielding the shape presented in Figure S15.^107^

The frequency-dependent refractive index (RI) of metallic Mg was taken from Palik,^108^ while the ambient, MgO, IPA and Si_3_N_4_ RIs were set to 1, 1.7, 1.3772, and 2.05, respectively. All calculations were carried out with MgO thicknesses as indicated, dipole distances of 1.0 nm, and a 2 nm gap between the NPs for dimers. A 20 nm thick Si_3_N_4_ substrate that extended at least 40 nm beyond the edges of the NP was used, when indicated, to account for the effect of the experimental TEM support film present in EELS measurements.

Scattering cross sections (*C*_sca_) were calculated as *Q*_sca_πα_eff_^2^, where *Q*_sca_ is the scattering efficiency taken directly from DDSCAT output, and α_eff_ the radius corresponding to a sphere of equal volume. Near-field enhancements (|*E*|/|*E*_0_|) were extracted from DDSCAT output via Paraview,^109^ for points one dipole away from the NP surface. The SERS EF was obtained by calculating (|*E*|/|*E*_0_|)^4^ at each of these points and subsequently averaging over the NP or NP dimer surface. Field-enhancement maps were plotted using Paraview such that they include the maximum field enhancement position on the NP surface. EEL probability maps were calculated in 5 nm steps in both directions and plotted using Matlab.

## Supporting Information Available

Additional SEM images, UV–vis/NIR spectra, STEM-EELS spectra, additional HAADF-STEM images for all NPs, additional STEM-EELS Mg bulk plasmon maps and STEM-EDS elemental maps for Mg NPs, all full Raman and SERS spectra, additional EF calculation tabulations, SERS spectra of Au NPs, ICP-OES results, NMF decomposition of STEM-EELS, eDDA point spectra, DDA scattering cross section plots, additional DDA electric field distribution maps, STEM-EDS line scan of Mg–Pd NPs, additional SERS evolution plots and maps of 4-NBT to DMAB conversion, modeled Mg NP shape, correlated dark field optical scattering spectroscopy, SERS, and SEM of Mg NPs (PDF)
