# Supplementary material for: Magnesium Nanoparticles for Surface-Enhanced Raman Scattering and Plasmon-Driven Catalysis
Source: ACS Nano. 2024 Jul 4;18(28):18785–99. doi: 10.1021/acsnano.4c06858 (PMC11256891; doi:10.1021/acsnano.4c06858)
Supplement: Supplementary file 1 — nn4c06858_si_001.pdf [file nn4c06858_si_001.pdf]

Supporting Information for

Magnesium Nanoparticles for Surface-Enhanced Raman Scattering and Plasmon-Driven Catalysis

Andrey Ten,[a,b] *Vladimir Lomonosov,*[a,b] *Christina Boukouvala,*[a,b] *and Emilie Ringe*[a,b]*

a. Department of Materials Science and Metallurgy, University of Cambridge, 27 Charles Babbage Road, Cambridge CB3 0FS, United Kingdom

b. Department of Earth Sciences, University of Cambridge, Downing Street, Cambridge CB2 3EQ, United Kingdom

* Corresponding Author: er407@cam.ac.uk

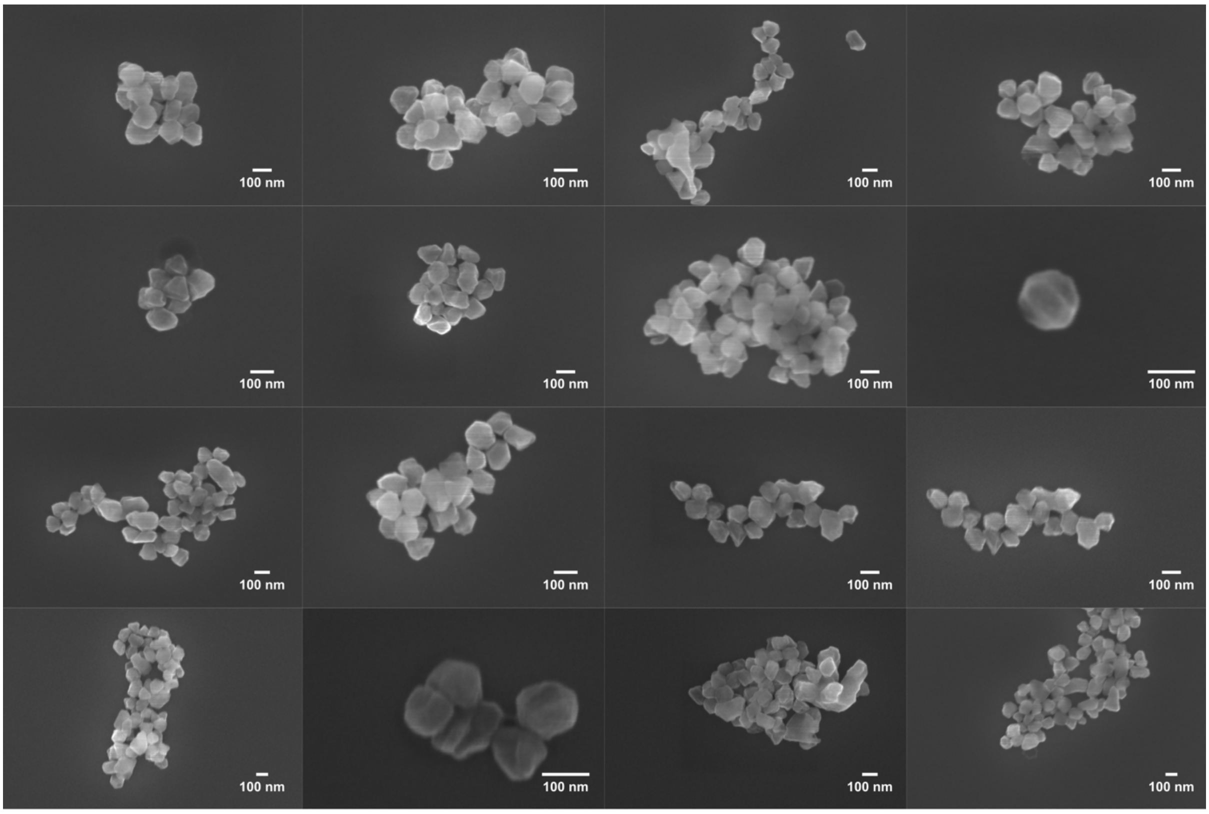

Figure S1. Additional SEM images of as-synthesized colloidal Mg faceted spheroids.

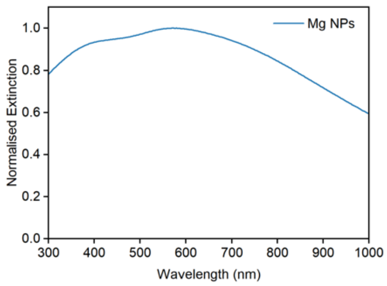

Figure S2. UV-Vis-NIR spectrum of as-synthesized colloidal Mg faceted spheroids with average size of 121 nm.

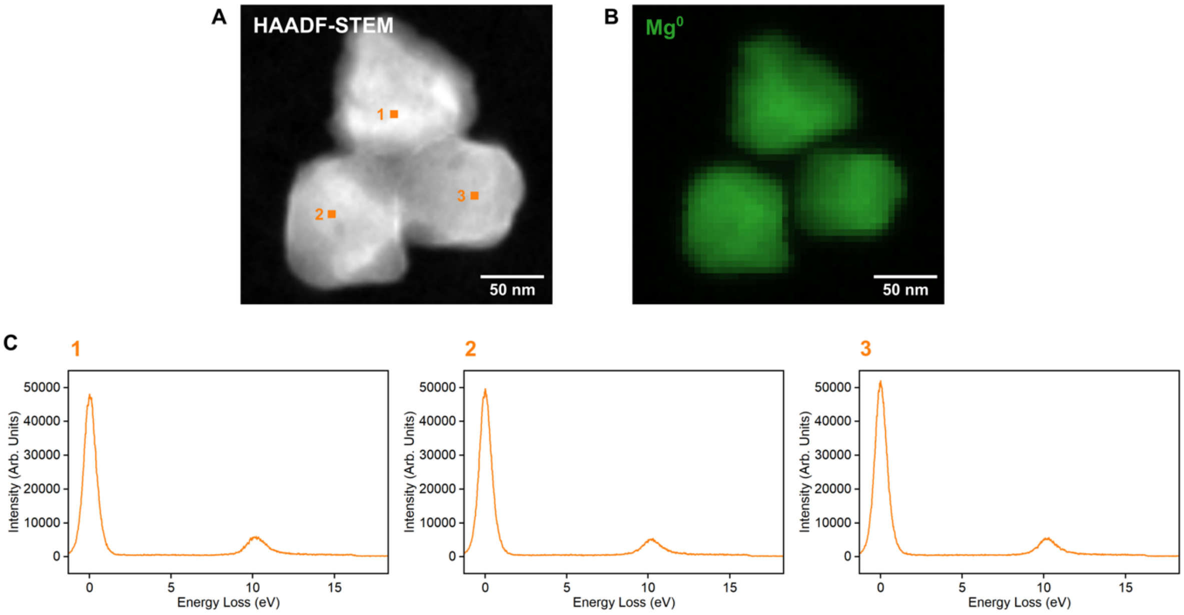

Figure S3. The STEM-EELS spectra showing the presence of the Mg bulk plasmon peak. (A) HAADF-STEM and (B) Mg bulk plasmon map shown in Figure 1, of Mg NPs post-incubation with 4-MBA. (C) STEM-EELS spectra from the positions labelled in A, showing the bulk plasmon peak of Mg at ~10.1 eV. The bulk plasmon map in B was produced by mapping the intensity of the bulk plasmon peak.

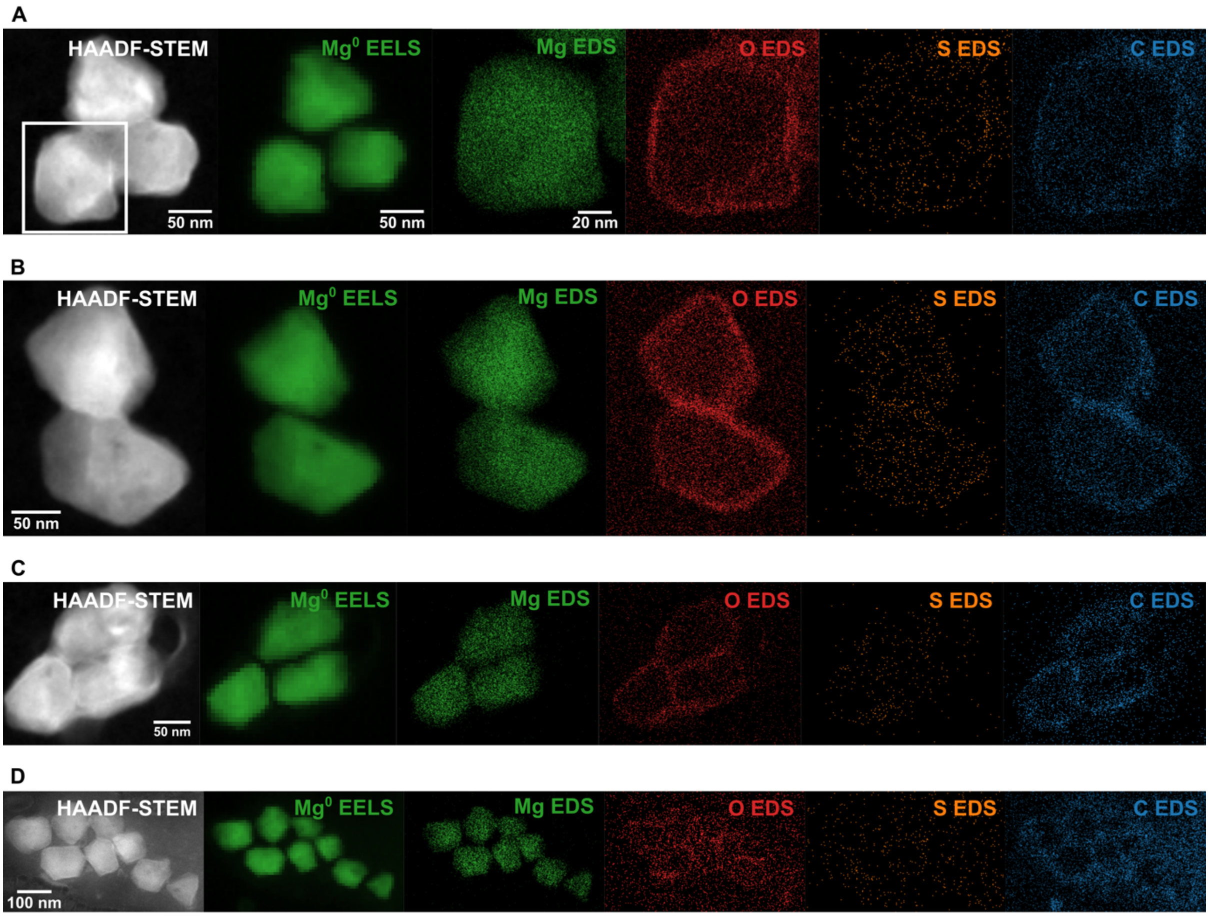

Figure S4. Additional HAADF-STEM, STEM-EELS Mg bulk plasmon maps, and STEM-EDS maps of Mg, O, S, and C for Mg NPs. (A – B) post-incubation with 4-MBA and (C – D) post-incubation with 4-NBT. The STEM-EDS maps in A are shown for the area marked with a white rectangle in the HAADF-STEM image.

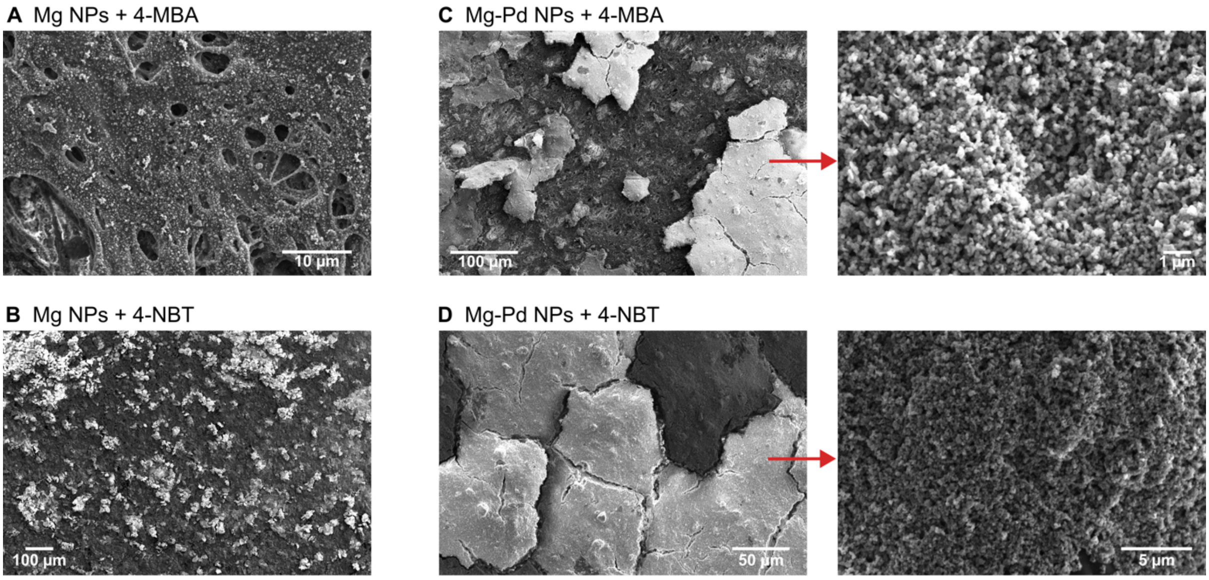

Figure S5. Mg and Mg-Pd NPs were successfully deposited on PES membrane filters. SEM images of deposited regions on filters for (A) Mg NPs post-incubation with 4-MBA, (B) Mg NPs post-incubation with 4-NBT, (C) Mg-Pd NPs post-incubation with 4-MBA, and (D) Mg-Pd NPs post-incubation with 4-NBT.

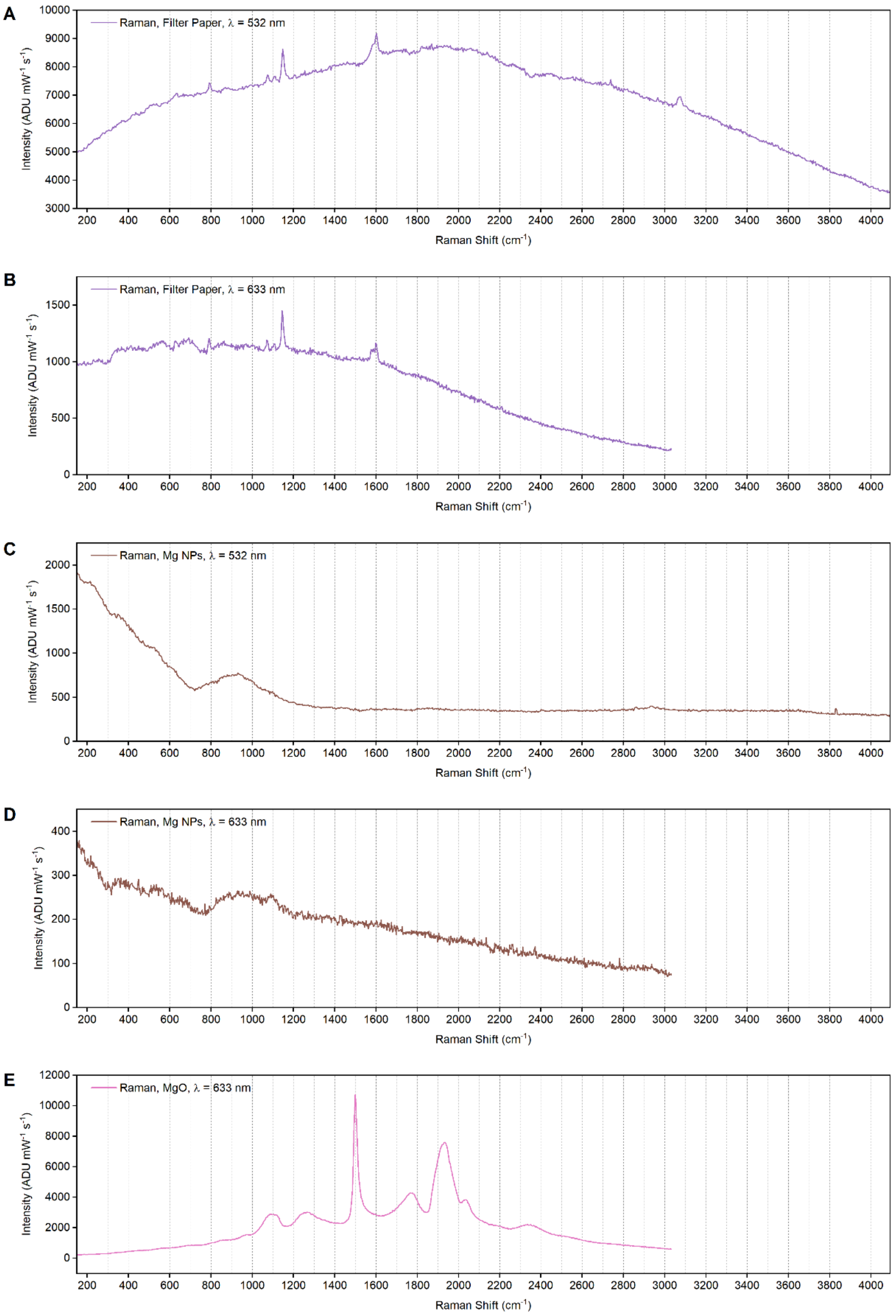

Figure S6. Normal Raman spectra of PES membrane filters acquired at (A) 532 and (B) 633 nm, of as-synthesized Mg NPs acquired at (C) 532 and (D) 633 nm, and of (E) MgO powder at 633 nm.

Vibrational Mode Assignment of SERS Spectra

The 4-MBA SERS spectra contain strong signals near 847 and 1417 cm^{-1}, assigned to COO^- (carboxylate anion) bending and symmetric stretching modes, respectively.[1–6] The presence of these peaks implies the formation of deprotonated carboxyl groups in 4-MBA, commonly observed on Au and Ag substrates in alkaline conditions or with low concentrations of 4-MBA.[1] In addition, the absence of the C=O stretching band near 1710 cm^{-1} confirms the dissociation of the carboxyl group,[1,3,4,6]

Using the Wilson scheme for benzene,[7] the bands at 1080 and 1593 cm^{-1} in 4-MBA SERS spectra are assigned to ν_{12} and ν_{9a} (often mislabeled as ν_{8a})[8] modes, respectively.[3,9,10] However, atomic motions in disubstituted benzene molecules such as 4-MBA differ from those defined by the Wilson scheme,[11] and therefore the Mulliken labeling[12] for asymmetric para-disubstituted benzene molecules with C_{2v} symmetry[8] (*e.g.*, deprotonated 4-MBA) is better suited. Using this labeling, the bands at 1080 and 1593 cm^{-1} are assigned to D_6 (containing C-S stretching) and D_3 modes, respectively.[8]

The dominant band in 4-NBT SERS spectra is the symmetric N-O stretching mode of the NO_2 group at 1335 cm^{-1},[13–16] at the same energy as in its normal Raman spectrum. The D_6 and D_3 modes of 4-NBT appear at 1080 and 1575 cm^{-1}, respectively,[13–16] close to those of 4-MBA owing to structural similarities. The peak at 848 cm^{-1} has previously been assigned to the D_{16} mode (containing C-H wagging).[13,16] The shoulder at 1295 cm^{-1} is assigned to the symmetric N-O stretching mode of the 4-NBT anion ($4\text{-}NBT^-$).

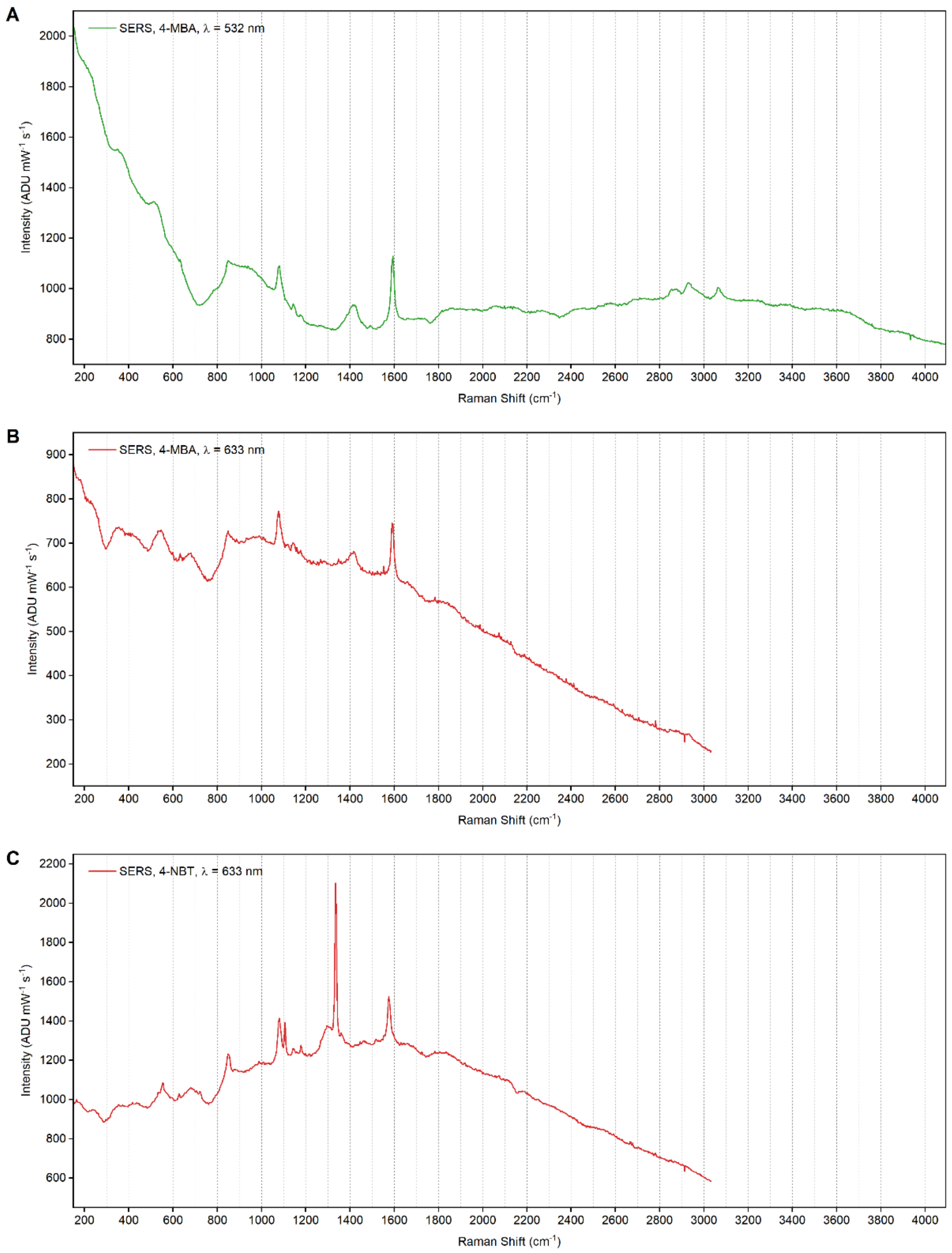

Figure S7. Full SERS spectra of analytes adsorbed on dry Mg NPs (partially shown in Figure 2), prior to background subtraction. (A) 4-MBA and 532 nm, (B) 4-MBA and 633 nm, and (C) 4-NBT and 633 nm. Each spectrum was averaged over 100 randomly selected acquisition regions.

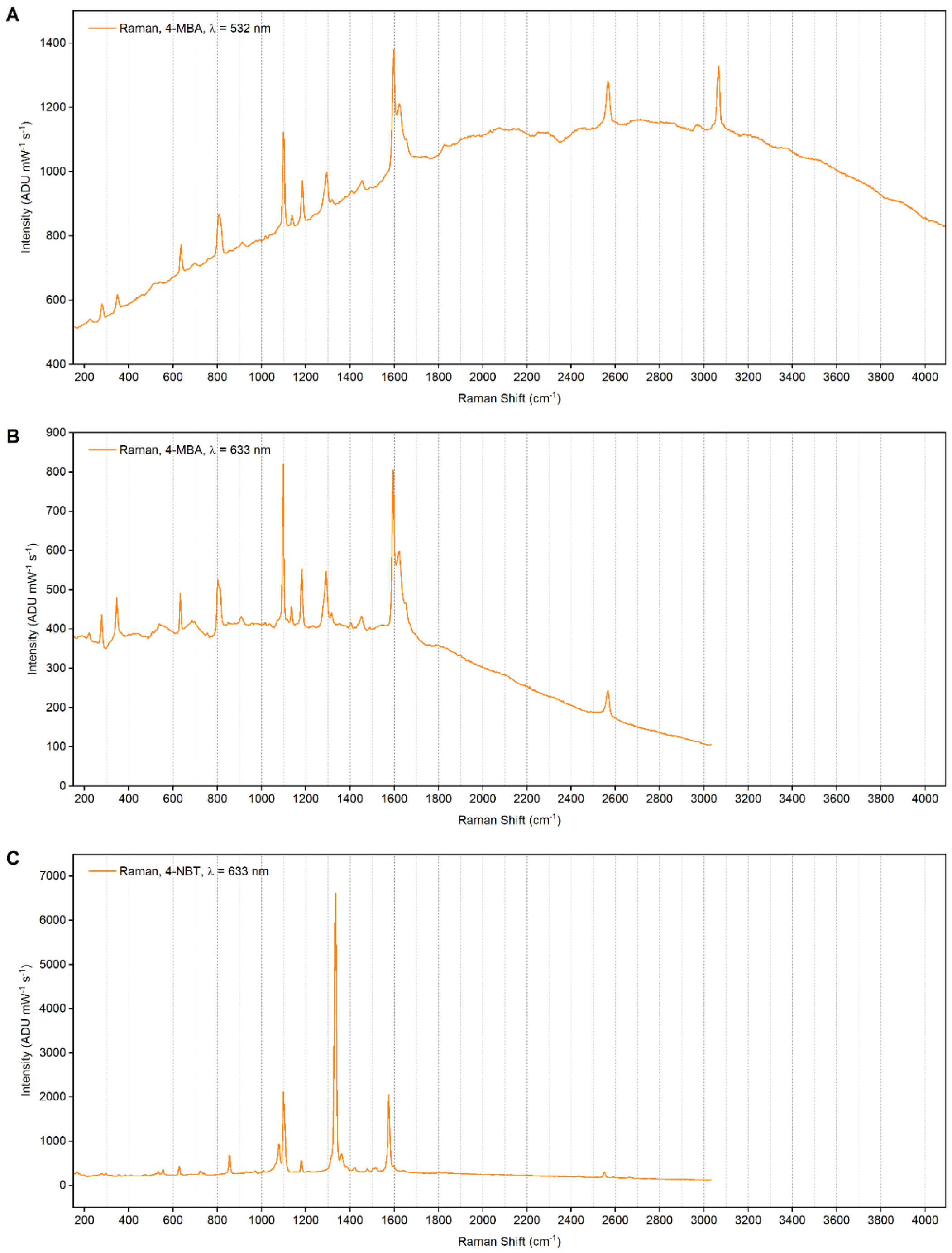

Figure S8. Full normal Raman spectra of analytes in solid form (partially shown in Figures 2 and 6), prior to background subtraction. The spectra are of analytes in their solid powdered form, where analyte and laser source are (A) 4-MBA and 532 nm, (B) 4-MBA and 633 nm, and (C) 4-NBT and 633 nm.

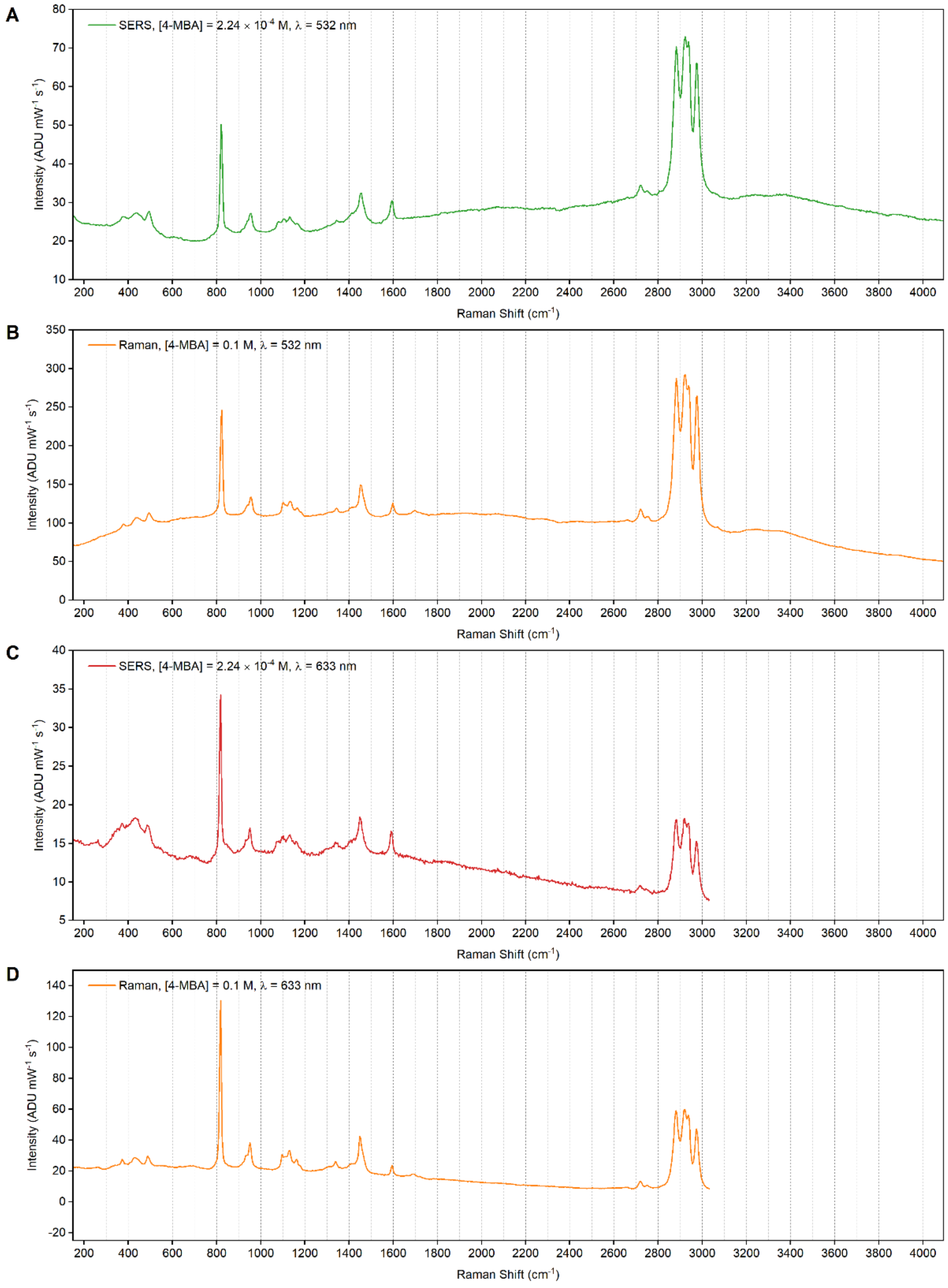

Figure S9. Full SERS spectra of 4-MBA-incubated colloidal Mg NPs dispersed in IPA and normal Raman spectra of 0.1 M 4-MBA solution in IPA (partially shown in Figure 3), prior to background subtraction. (A) SERS spectra (B) normal Raman spectra at 532 nm. (C) SERS spectra (D) normal Raman spectra at 633 nm. The 4-MBA concentration in SERS were quantified with ICP-OES (Table S1). Each spectrum was averaged over 10 acquisitions. The spectra include contributions from IPA.

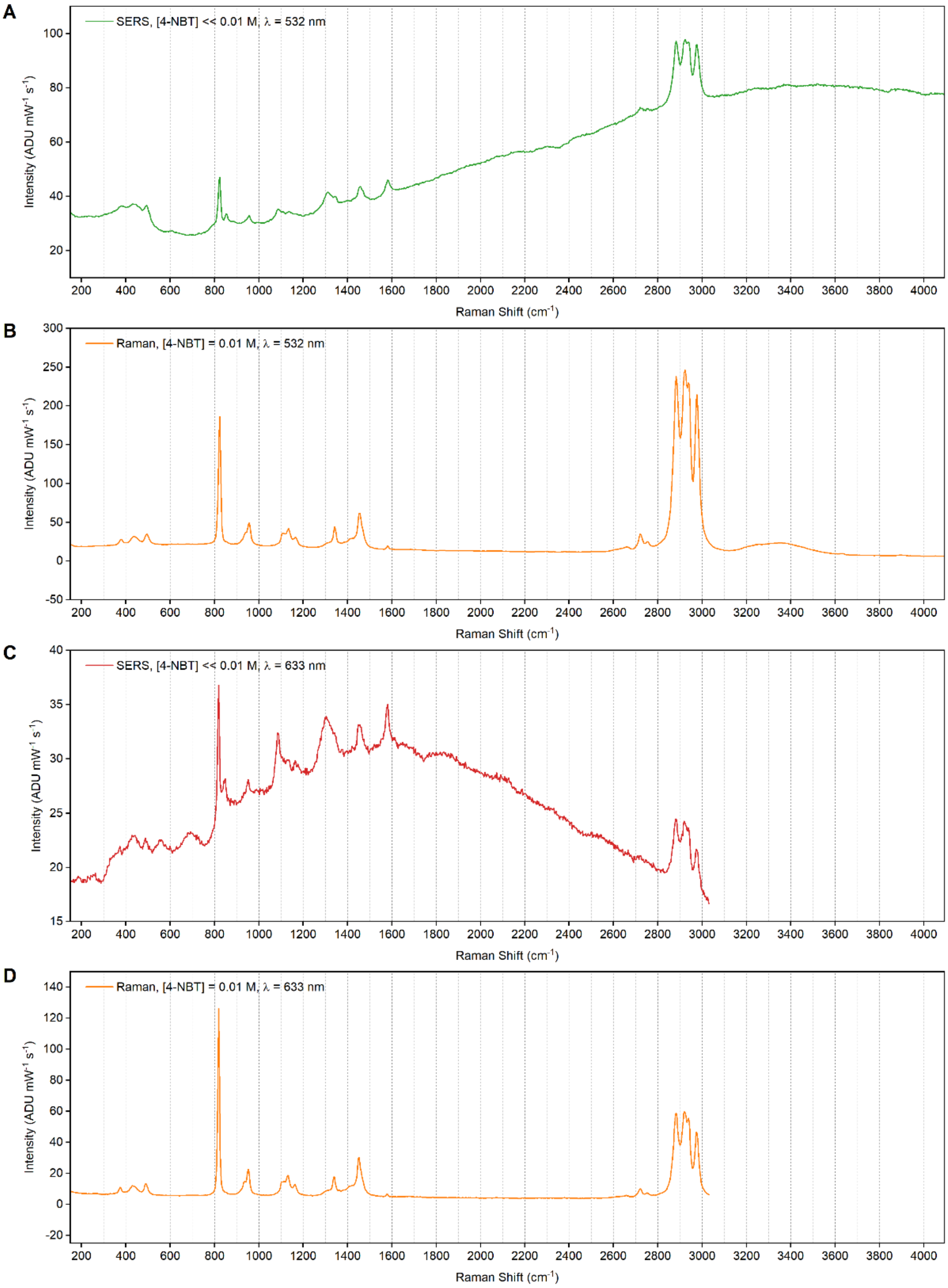

Figure S10. Full SERS spectra of 4-NBT-incubated colloidal Mg NPs dispersed in IPA and normal Raman spectra of 0.01 M 4-NBT solution in IPA (partially shown in Figure 3), prior to background subtraction. (A) SERS spectra (B) normal Raman spectra at 532 nm. (C) SERS spectra (D) normal Raman spectra at 633 nm. The 4-NBT concentration in SERS are estimated from the incubation solution concentration and subsequent cleaning steps. Each spectrum was averaged over 10 acquisitions. The spectra include contributions from IPA.

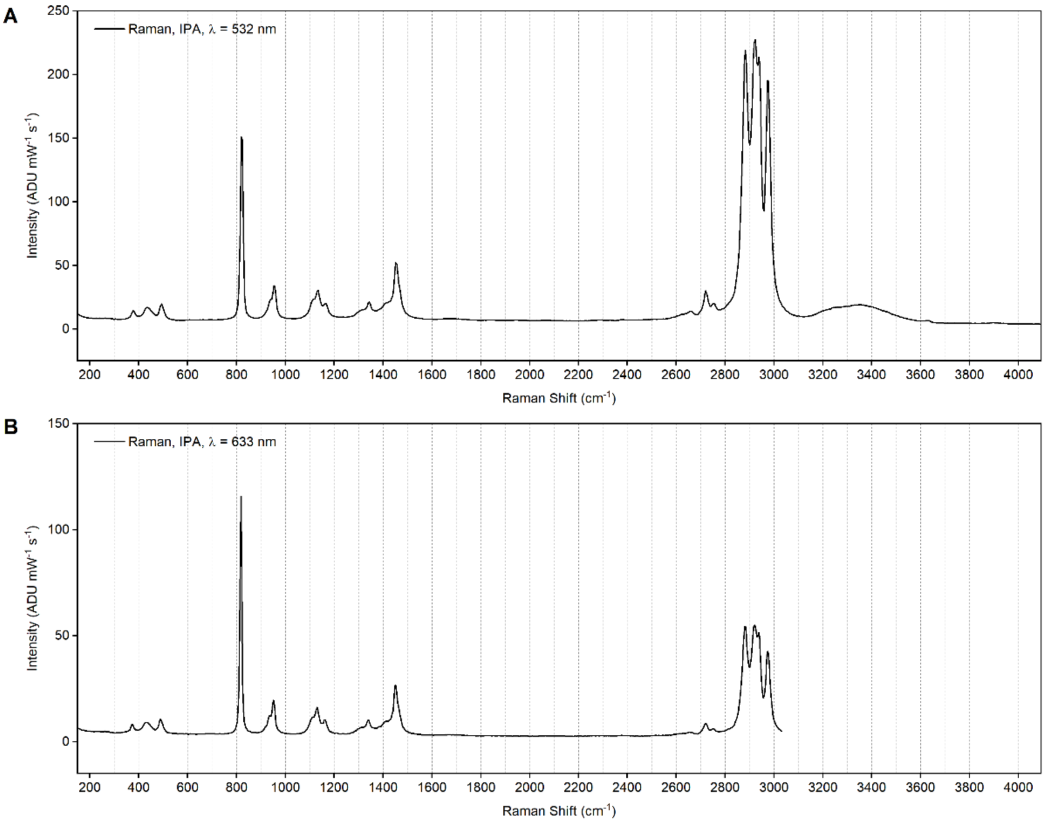

Figure S11. Full normal Raman spectra of IPA at (A) 532 (B) a 633 nm.

Table S1. ICP-OES Results

Sample	Element	Concentration (mg/L)
Mg NPs with 4-MBA	Mg	1280
	S	7.19
Mg NPs with 4-NBT	Mg	1236
Mg-Pd NPs with 4-MBA	Mg	718
	Pd	93.75
	S	36.4
Mg-Pd NPs with 4-NBT	Mg	910
	Pd	154
Au NPs with 4-MBA	Au	413.4
	S	1.57

**Table S2.** EFs of Mg NPs calculated using N_{Surf} obtained by S content in ICP-OES.		
Analyte	4-MBA	
Laser Wavelength (nm)	532	633
I_{SERS} (ADU mW^{-1} s^{-1})	5.44	3.04
I_{Raman} (ADU mW^{-1} s^{-1})	15.80	6.19
$N_{Surf,}$ S ICP-OES (molecules)	8.83 x 10^6	
N_{Vol} (molecules)	3.94 x 10^9	
EF, S ICP-OES	154	219

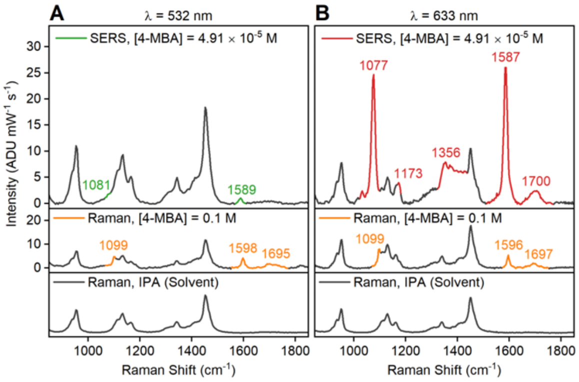

Figure S12. SERS spectra of 4-MBA-incubated colloidal spherical Au NPs dispersed (58 nm in diameter) in IPA. The spectra were collected at (A) 532 and (B) 633 nm. The normal Raman spectra of 0.1 M 4-MBA solution in IPA and the spectra of IPA are shown as a reference, under the SERS spectra. The spectral features of IPA are plotted in black while the peaks from 4-MBA are highlighted in color. The 4-MBA concentration in SERS were quantified with ICP-OES (Table S1).

**Table S3.** EFs of 58 nm spherical Au NPs		
Analyte	4-MBA	
Laser Wavelength (nm)	532	633
I_{SERS} (ADU mW^{-1} s^{-1})	1.04	26.04
I_{Raman} (ADU mW^{-1} s^{-1})	4.27	5.49
$N_{Surf,}$ S ICP-OES (molecules)	1.93 x 10^6	
$N_{Surf,}$ Monolayer Estimation (molecules)	2.72 x 10^7	
N_{Vol} (molecules)	3.94 x 10^9	
EF, S ICP-OES	496	9658
EF, Monolayer Estimation	35	686

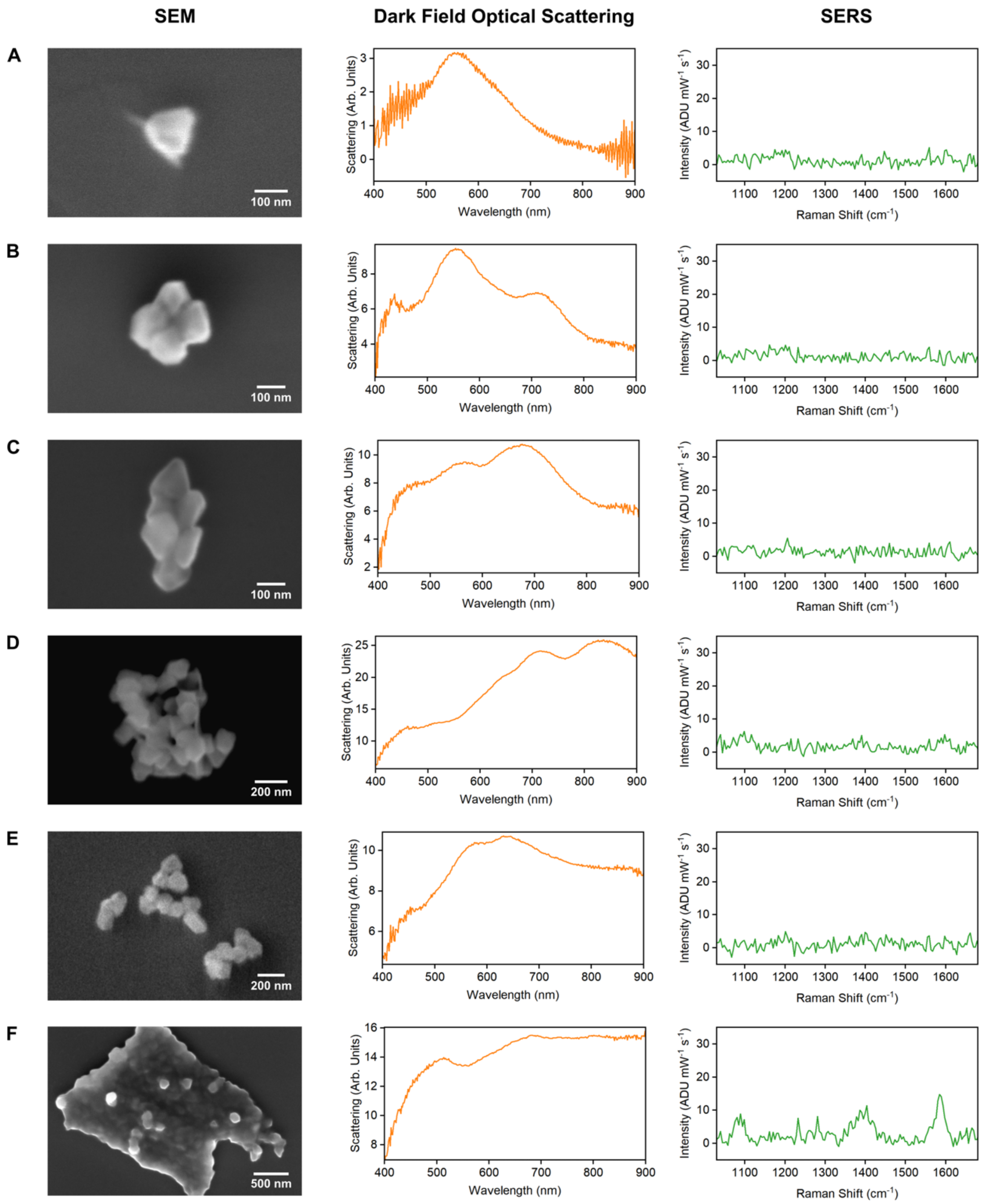

Figure S13. Correlated SEM, dark field optical scattering spectroscopy, and SERS of 4-MBA-bound Mg NPs. The region of interest consists of (A) a single particle and (B – F) aggregates. The SERS spectra were collected at 532 nm.

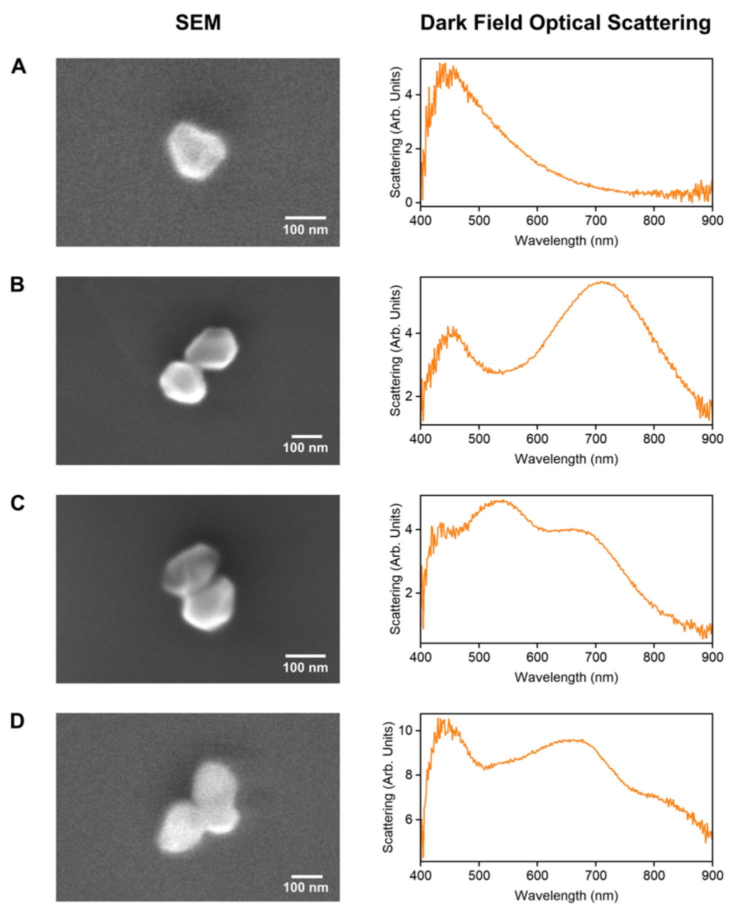

Figure S14. Correlated SEM and dark field optical scattering spectroscopy of 4-MBA-bound Mg NPs. The region of interest consists of (A) a single particle, (B – C) a dimer, and (D) a trimer.

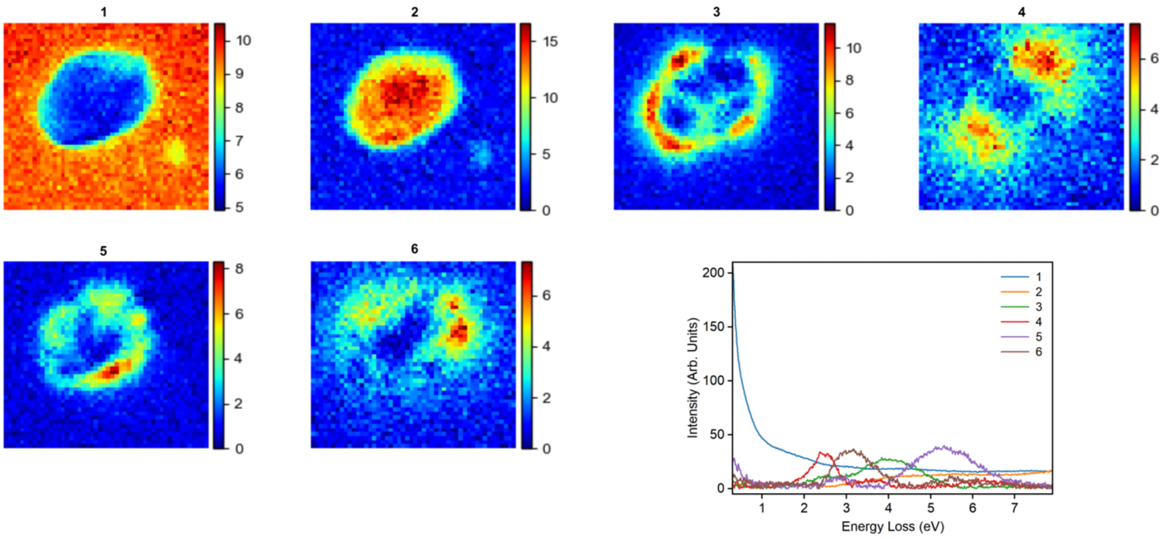

Figure S15. NMF results on a single Mg faceted spheroid, for the STEM-EELS data presented in Figure 4. NMF spatial loadings and their corresponding spectral factors (bottom right) are extracted. Component 4 is responsible for the dipole mode found in the Mg NP monomer.

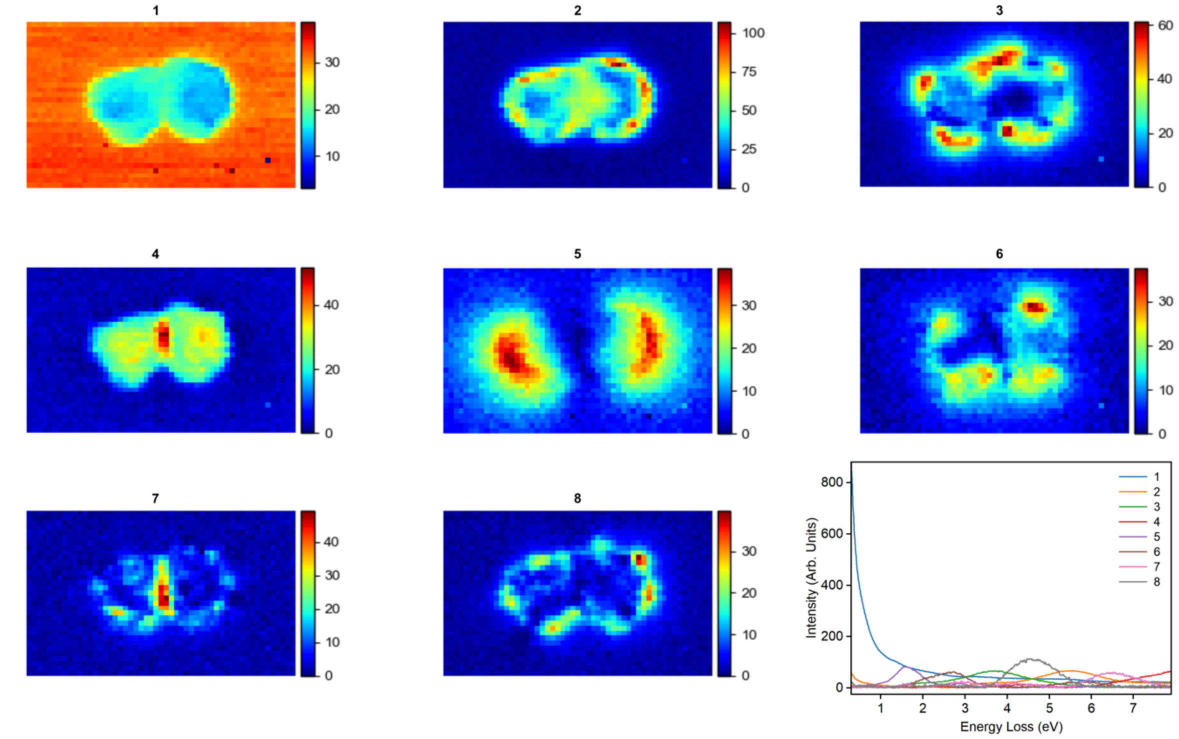

Figure S16. NMF results on a dimer of Mg faceted spheroids, for the STEM-EELS data presented in Figure 5. NMF spatial loadings and their corresponding spectral factors (bottom right) are extracted. Decomposition 5 depicts the loss probability of the bonding mode found in the Mg NP dimer.

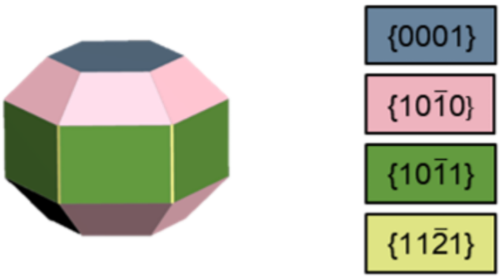

Figure S17. The Wulff-constructed shape of a Mg NP used for DDA and e-DDA simulations.

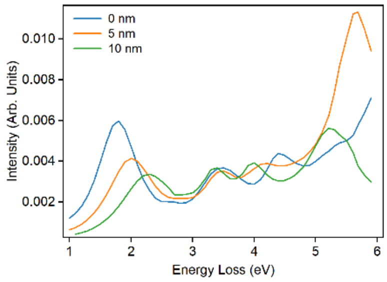

Figure S18. The e-DDA point spectrum of a modelled Mg NP dimer of varying oxide thicknesses (0, 5, and 10 nm), calculated at the tip of the dimer as indicated by a red box in Figure 5A. The tip-to-tip length of the NPs including the oxide layer was kept at 120 nm.

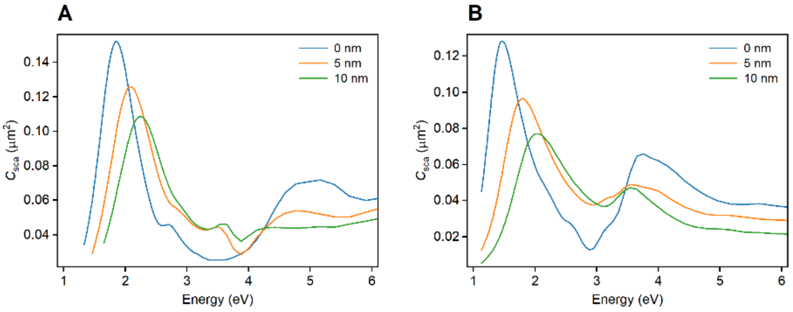

Figure S19. Additional DDA scattering cross sections (C_{sca}) of a modeled Mg NP dimer positioned facet-to-facet with a 2 nm interparticle gap of varying oxide thicknesses (0, 5, and 10 nm) (A) rested on a 20 nm Si_3N_4 layer and (B) in IPA medium. The tip-to-tip length of the NPs including the oxide layer was kept at 120 nm.

**Table S4.** Calculated average electromagnetic EFs of a Mg NP dimer with varying oxide layer thickness, using $|E(\omega)|^4/|E_0|^4$ and $|E(\omega)|^2/|E_0|^2 \times |E(\omega')|^2/|E_0|^2$, where ω and ω' are maximum Q_{sca} and stokes-shifted (1593 cm^{-1}) frequencies, respectively.			
Oxide layer thickness (nm)	Maximum Q_{sca} (ω, nm)	EF, $\|E(\omega)\|^4/\|E_0\|^4$	EF, $\|E(\omega)\|^2/\|E_0\|^2 \times \|E(\omega')\|^2/\|E_0\|^2$
0	850	101712	99092
5	690	1951	2274
10	610	318	382

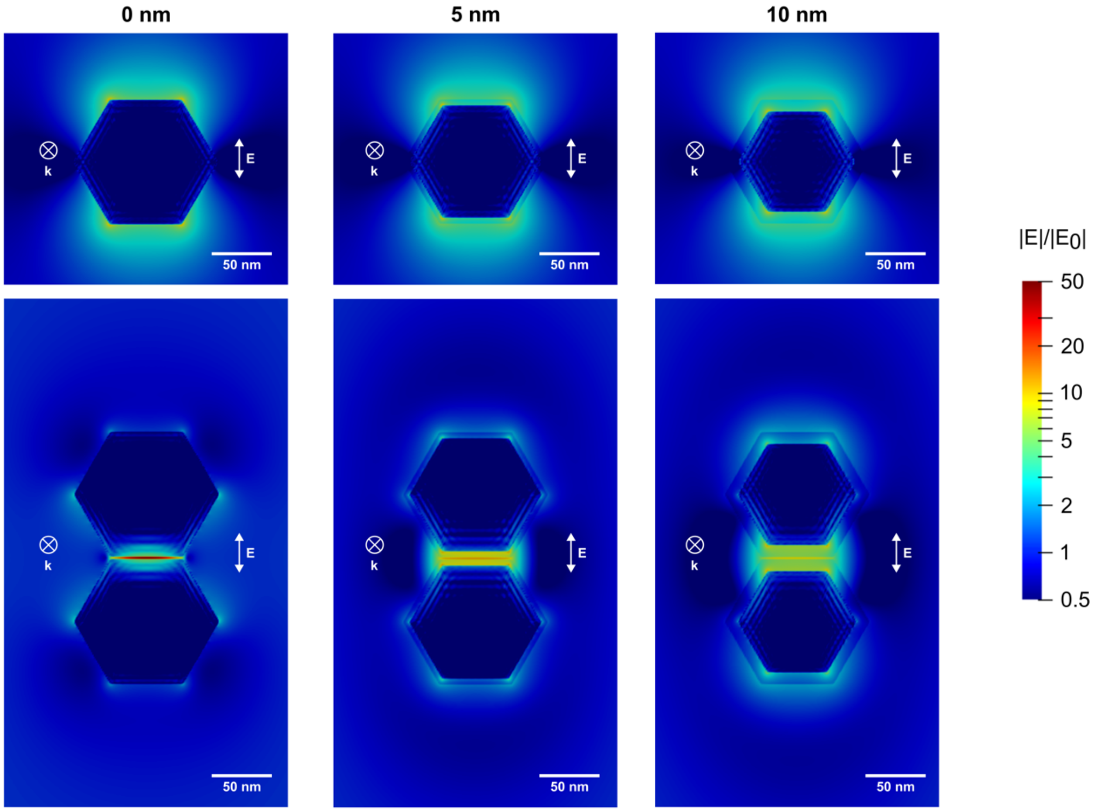

Figure S20. The DDA electric field distribution maps of monomers (top) and dimers of NPs placed 2 nm apart along their facets (bottom) consisting of Wulff-constructed Mg NPs with varying oxide thickness (0, 5, 10 nm) in IPA medium. The tip-to-tip length of the NPs including the oxide layer was kept at 120 nm and the maps were plotted at 532 nm.

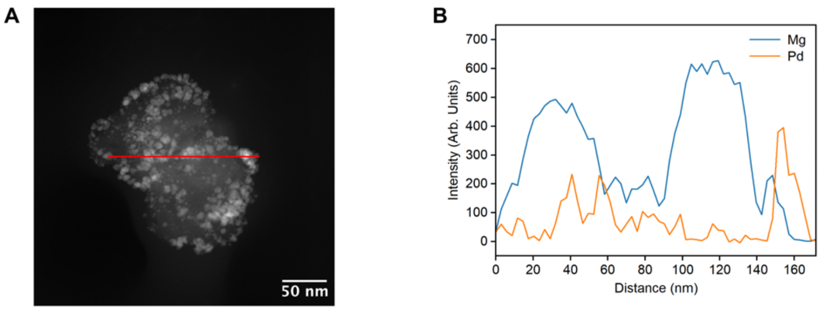

Figure S21. Detailed measurement of atomic composition of a Mg-Pd NP from STEM-EDS. (A) HAADF-STEM image of NPs through which a line scan was collected (red line). (B) The spatial distribution of Mg and Pd along the line.

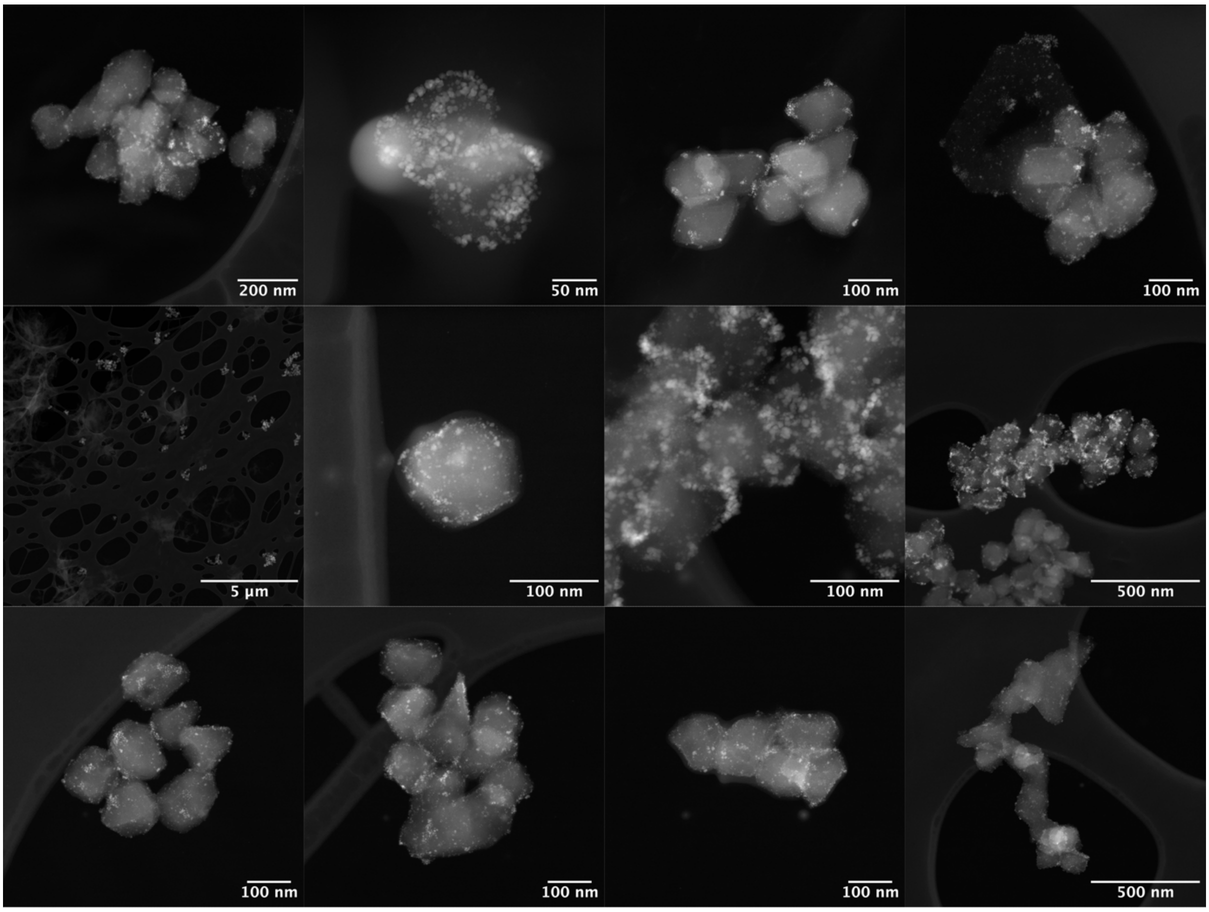

Figure S22. Additional HAADF-STEM images of Mg-Pd NPs. The bright spots correspond to Pd.

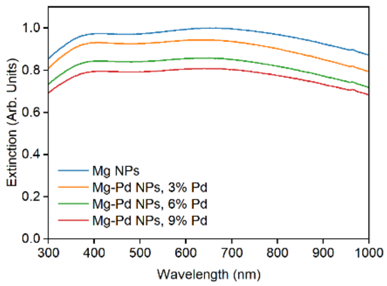

Figure S23. UV-Vis-NIR spectrum of colloidal Mg faceted spheroids before and after galvanic replacement with Pd using 3, 6 and 9 mol %.

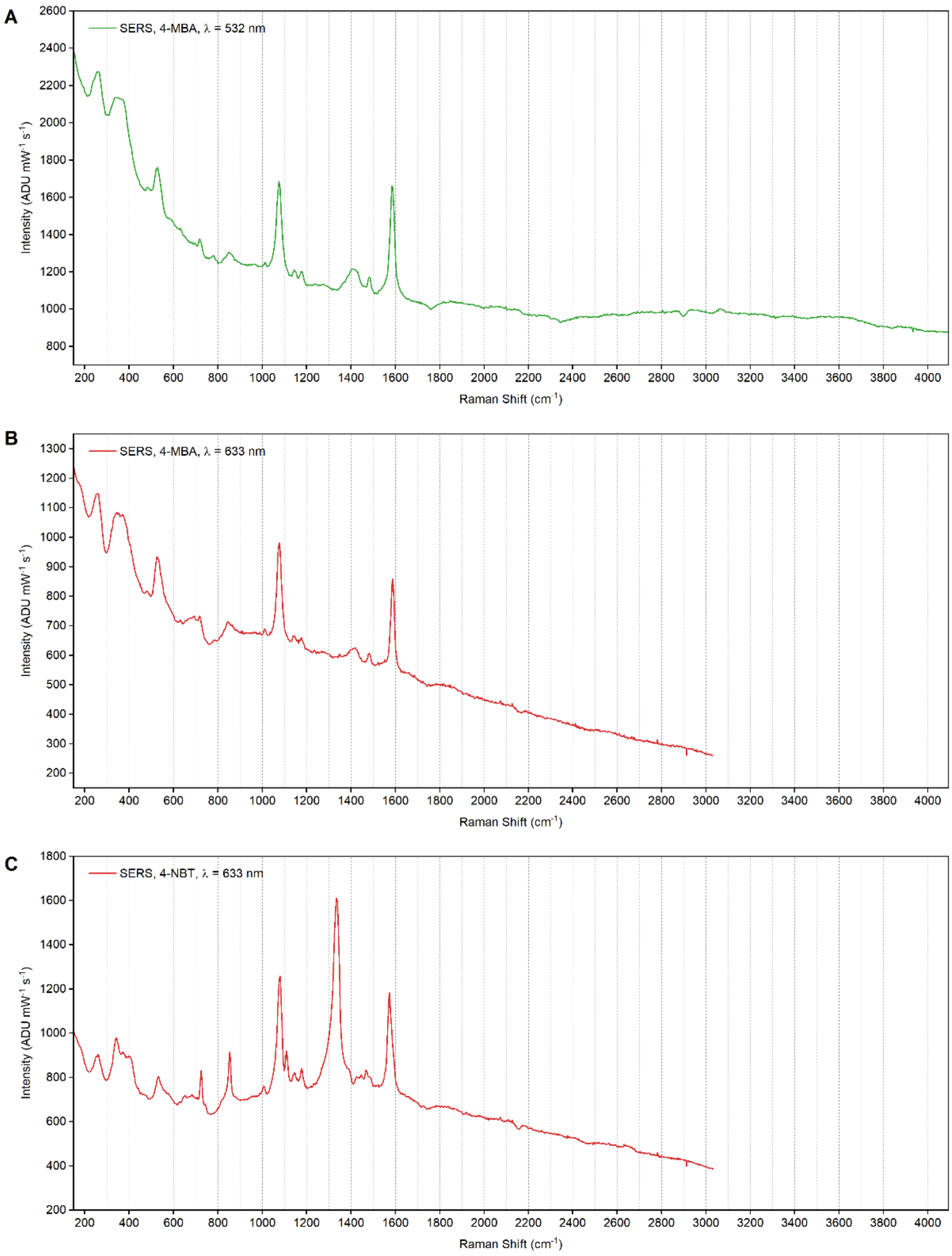

Figure S24. Full SERS spectra of analytes adsorbed on dry Mg-Pd NPs (partially shown in Figure 6), prior to background subtraction. (A) 4-MBA and 532 nm, (B) 4-MBA and 633 nm, and (C) 4-NBT and 633 nm. Each spectrum was averaged over 100 randomly selected acquisition regions.

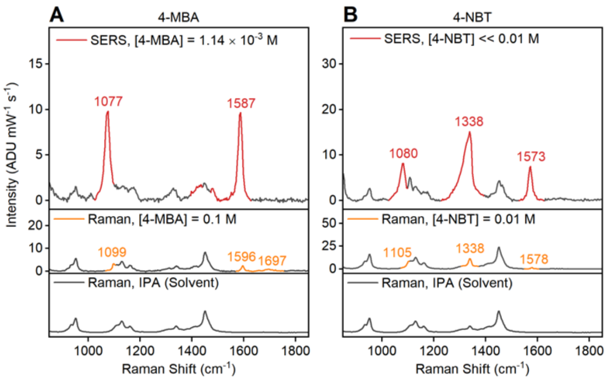

Figure S25. SERS spectra of analyte-incubated colloidal Mg-Pd NPs dispersed in IPA. (A) 4-MBA and (B) 4-NBT was used at 633 nm. The normal Raman spectra of 0.1 M 4-MBA and 0.01 M 4-NBT solution in IPA, as well as the spectra of IPA are shown as a reference, under the SERS spectra. The spectral features of IPA are plotted in black while the peaks from analytes are highlighted in color. The 4-MBA concentration in SERS were quantified with ICP-OES (Table S1). The 4-NBT concentration in SERS are estimated from the incubation solution concentration and subsequent cleaning steps. Full raw spectra without background subtraction are reported in Figures S26, S27, and S11.

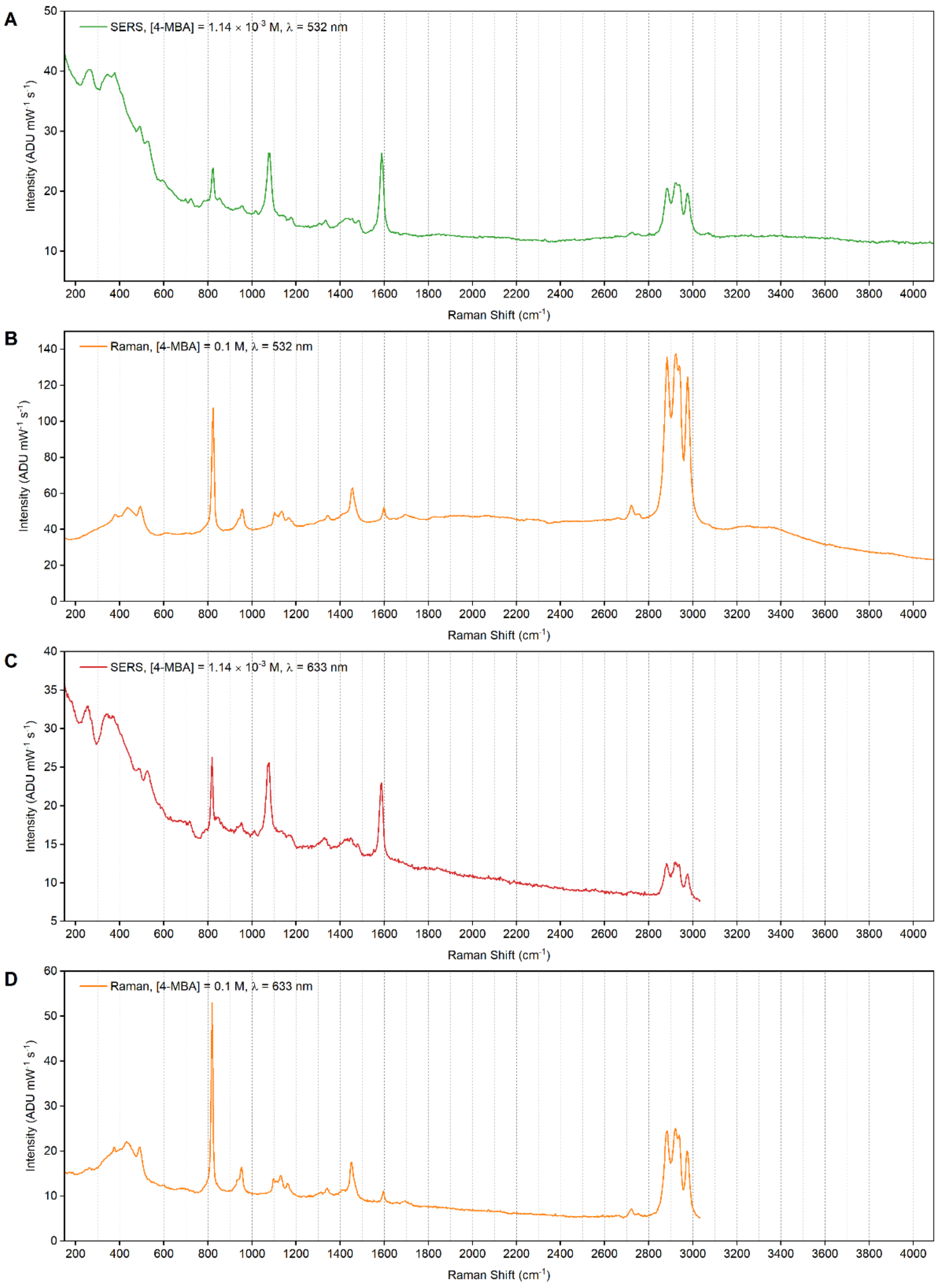

Figure S26. Full SERS spectra of 4-MBA-incubated colloidal Mg-Pd NPs dispersed in IPA and normal Raman spectra of 0.1 M 4-MBA solution in IPA (partially shown in Figures 7 and S25), prior to background subtraction. (A) SERS and (B) normal Raman spectra at 532 nm. (C) SERS and (D) normal Raman spectra at 633 nm. The 4-MBA concentration in SERS were quantified with ICP-OES (Table S1). Each spectrum was averaged over 10 acquisitions. The spectra include contributions from IPA.

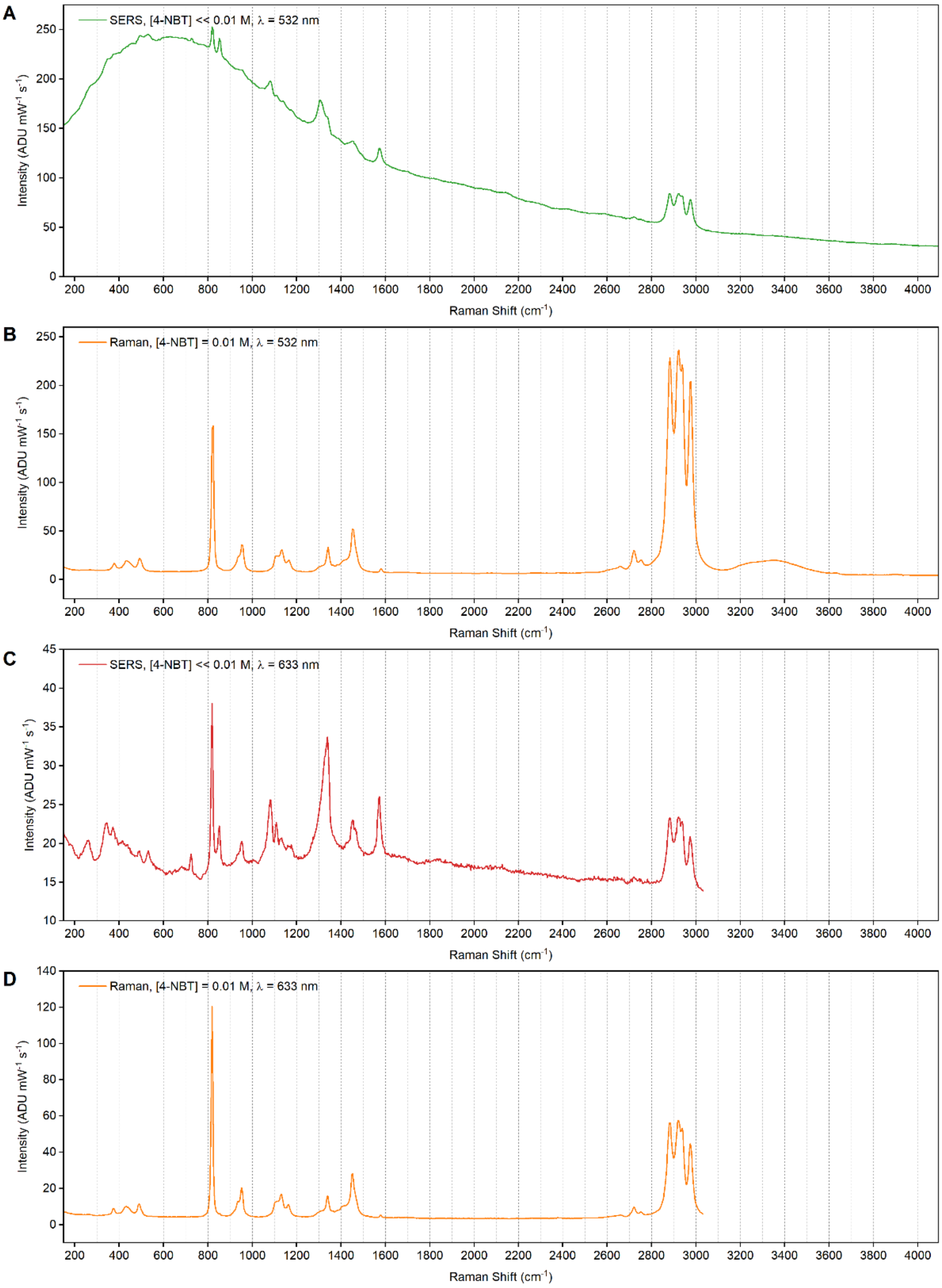

Figure S27. Full SERS spectra of 4-NBT-incubated colloidal Mg NPs dispersed in IPA and normal Raman spectra of 0.01 M 4-NBT solution in IPA (partially shown in Figures 7 and S25), prior to background subtraction. (A) SERS and (B) normal Raman spectra at 532 nm. (C) SERS and (D) normal Raman spectra at 633 nm. The 4-NBT concentration in SERS are estimated from the incubation solution concentration and subsequent cleaning steps. Each spectrum was averaged over 10 acquisitions. The spectra include contributions from IPA.

Alternative approach to calculating the surface area of Mg-Pd NPs

An alternative way to calculate the total surface area is to assume that Mg-Pd NPs are formed of 121 nm Mg spheres with 5 nm Pd spheres embedded on the surface of Mg spheres. We further assume that half the surface area of each Pd sphere is available. The calculation employs Mg and Pd concentrations from ICP-OES (Table S1), as was done for the monolayer approximation calculation in the main text assuming 121 nm spheres for both Mg and Pd. Compared to the monolayer approximation with 121 nm spheres, the resulting EF values are 20 and 26 % lower for 4-MBA and 4-NBT, respectively (Table S5).

Table S5. Enhancement factors (EFs) of Mg-Pd NPs calculated using N_{Surf} obtained by monolayer estimation on 121 nm Mg spheres embedded with 5 nm Pd spheres.

Analyte	4-MBA		4-NBT	
Laser Wavelength (nm)	532	633	532	633
I_{SERS} (ADU mW^{-1} s^{-1})	13.27	9.65	16.06	7.44
I_{Raman} (ADU mW^{-1} s^{-1})	7.19	2.45	3.74	1.39
N_{Surf} (molecules)	7.48 x 10^{6}		1.00 x 10^{7}	
N_{Vol} (molecules)	3.94 x 10^{9}		3.94 x 10^{8}	
EF, S ICP-OES	971	2072	169	211

Table S6. Enhancement factors (EFs) of Mg-Pd NPs calculated using N_{Surf} obtained by S content in ICP-OES.

Analyte	4-MBA	
Laser Wavelength (nm)	532	633
I_{SERS} (ADU mW^{-1} s^{-1})	13.27	9.65
I_{Raman} (ADU mW^{-1} s^{-1})	7.19	2.45
$N_{Surf,}$ S ICP-OES (molecules)	4.47 x 10^{7}	
N_{Vol} (molecules)	3.94 x 10^{9}	
EF, S ICP-OES	162	347

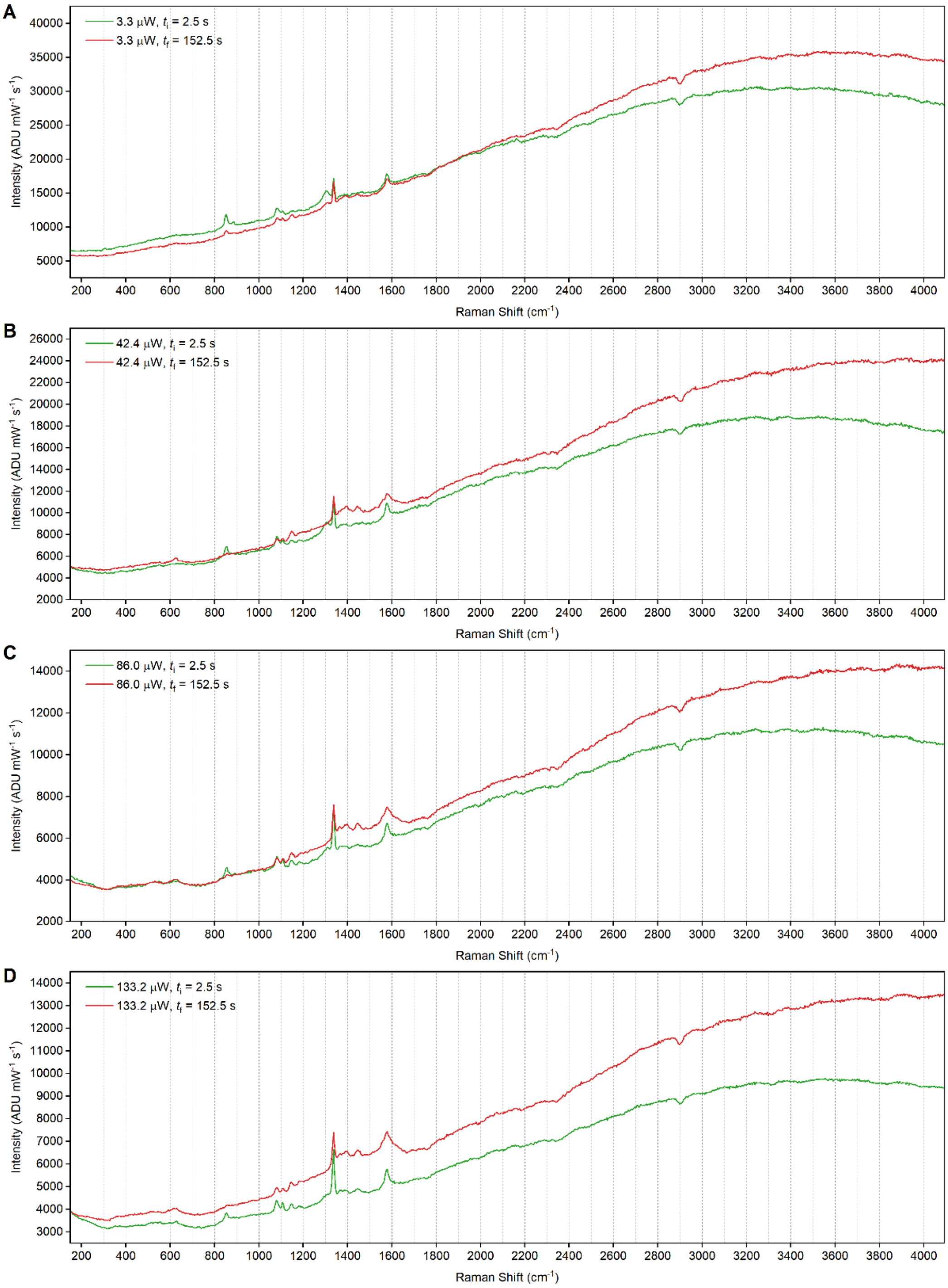

Figure S28. Full SERS spectra of 4-NBT on dry Mg NPs at t_i = 2.5 s and t_f = 152.5 s upon 532 nm excitation at (A) 3.3, (B) 42.4, (C) 86.0, and (D) 133.2 µW laser power (partially shown in Figure 8), prior to background subtraction. The spectra at t_i = 2.5 s and t_f = 152.5 s are shown in green and red, respectively.

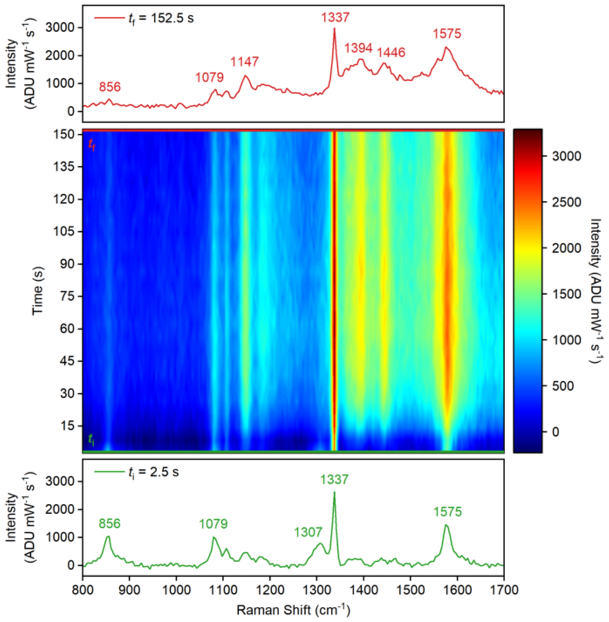

Figure S29. Additional evolution map of SERS spectra of 4-NBT on dry Mg NPs over time upon 532 nm excitation at 42.4 μW laser power. The spectra were averaged across 20 acquisitions and the background at t_i = 2.5 s was subtracted across all subsequent spectra. The spectra at t_i = 2.5 s and t_f = 152.5 s are shown in green and red, respectively.

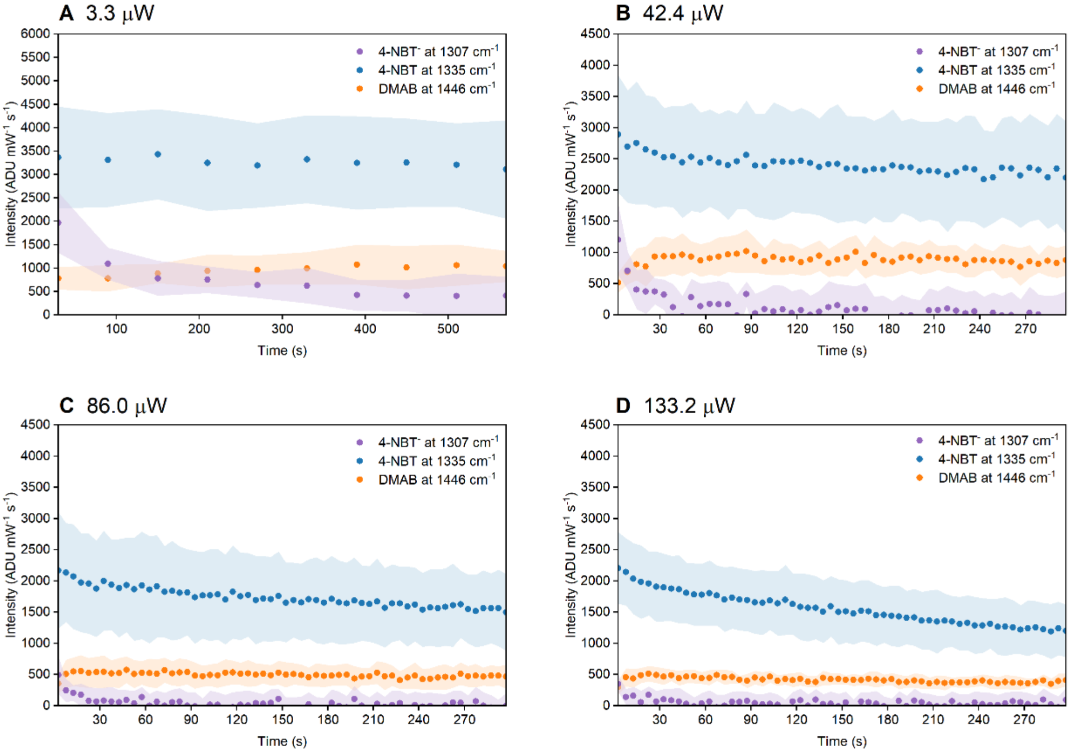

Figure S30. Additional plots showing the change in SERS peak intensities of 4-NBT on dry Mg NPs over time 532 nm excitation at (A) 3.3, (B) 42.4, (C) 86.0, and (D) 133.2 μW laser power. The peak intensities are shown for the N-O stretching modes at 1307 and 1337 cm^{-1} and the -N=N- stretching mode at 1446 cm^{-1} from 4-NBT^{-}, 4-NBT, and DMAB, respectively. The data points represent the average peak intensities and the colored background represent their standard deviation. The plotted time represents the midpoint of each back-to-back acquisition.

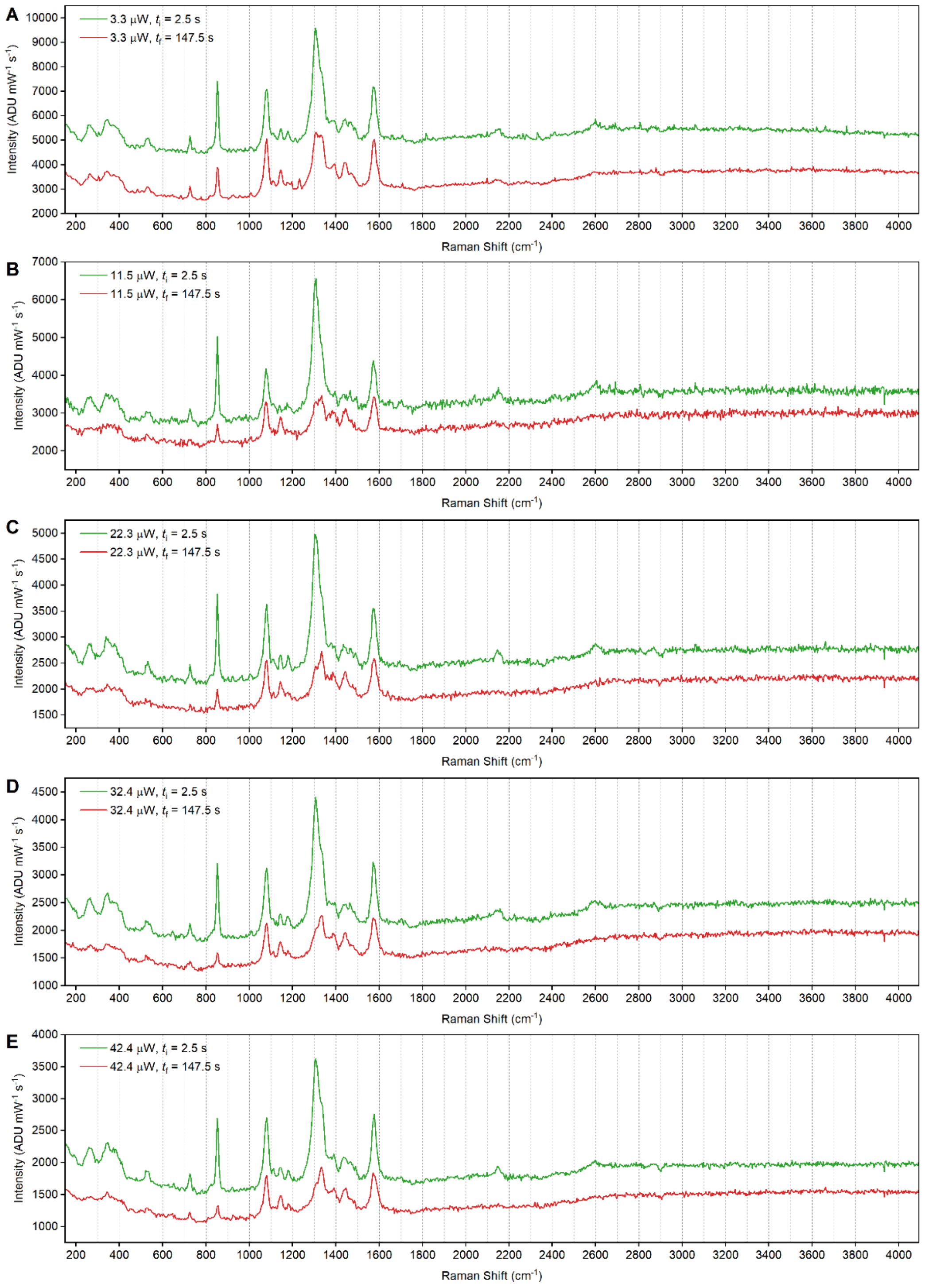

Figure S31. Full SERS spectra of 4-NBT on dry Mg-Pd NPs at t_i = 2.5 s and t_f = 147.5 s upon 532 nm excitation at (A) 3.3, (B) 11.5, (C) 22.3, (D) 32.4, and (E) 42.4 µW laser power (partially shown in Figure 8), prior to background subtraction. The spectra at t_i = 2.5 s and t_f = 147.5 s are shown in green and red, respectively.

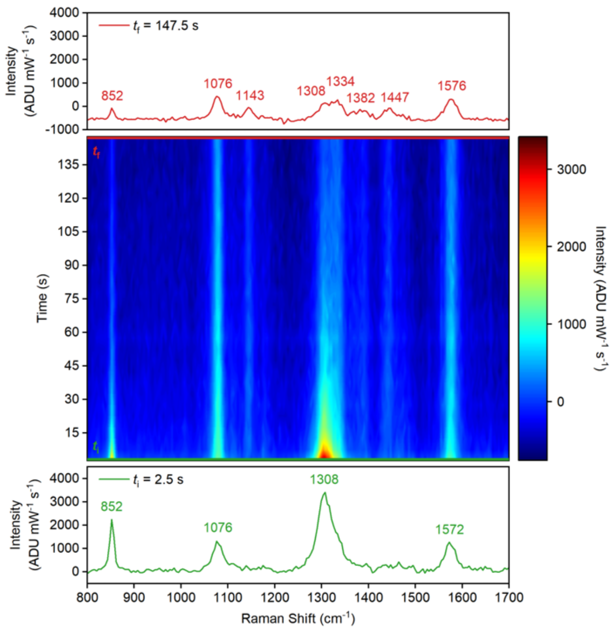

Figure S32. Additional evolution map of SERS spectra of 4-NBT on dry Mg-Pd NPs over time upon 532 nm excitation at 11.5 µW laser power. The spectra were averaged across 20 acquisitions and the background at t_i = 2.5 s was subtracted across all subsequent spectra. The spectra at t_i = 2.5 s and t_f = 147.5 s are shown in green and red, respectively.

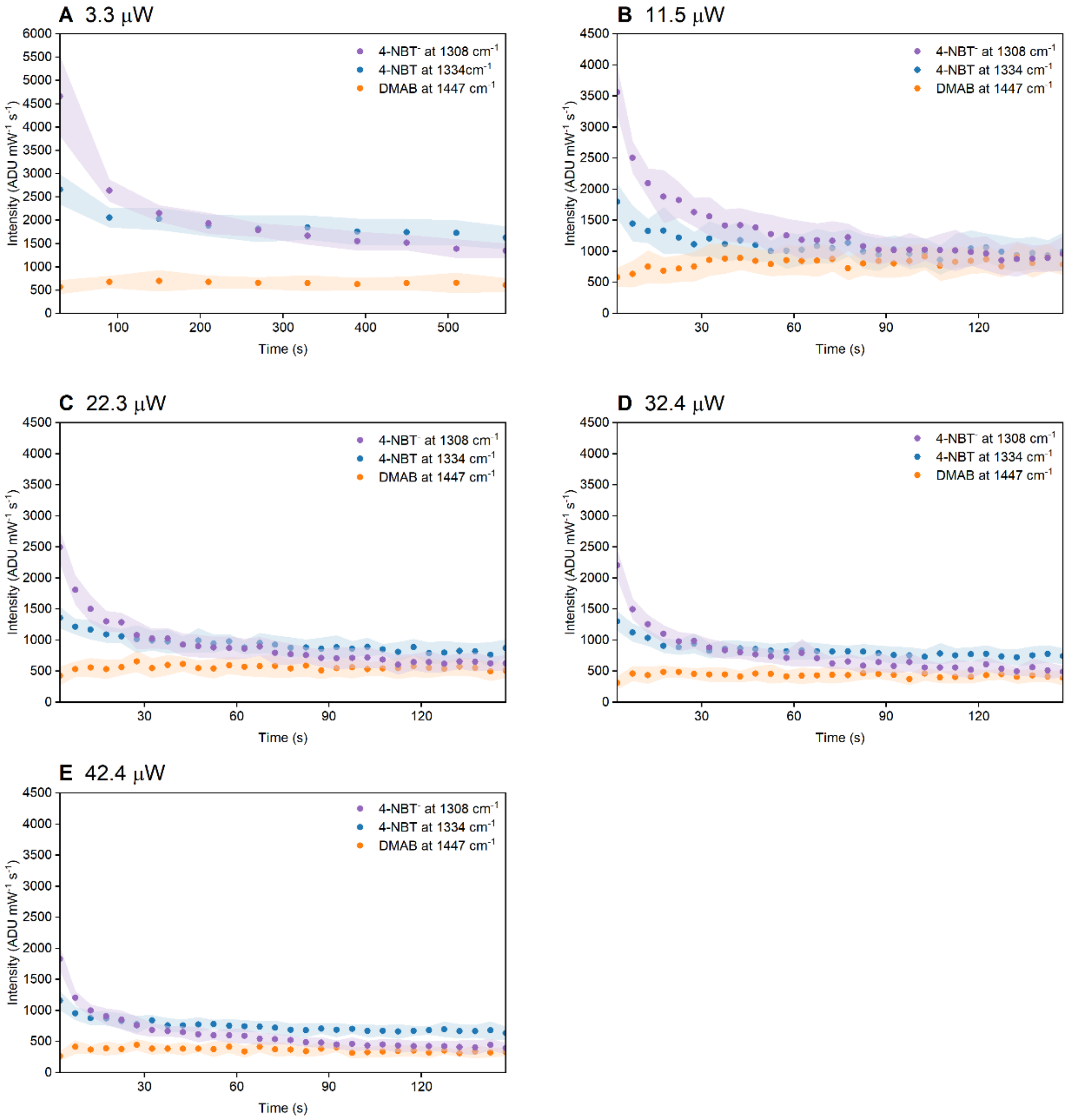

Figure S33. Additional plots showing the change in SERS peak intensities of 4-NBT on dry Mg-Pd NPs over time upon 532 nm excitation at (A) 3.3, (B) 11.5, (C) 22.3, (D) 32.4, and (E) 42.4 µW laser power. The peak intensities are shown for the N-O stretching modes at 1308 and 1334 cm^{-1} and the -N=N- stretching mode at 1447 cm^{-1} from 4-NBT^{-}, 4-NBT, and DMAB, respectively. The data points represent the average peak intensities and the colored background represent their standard deviation. The plotted time represents the midpoint of each back-to-back acquisition.

REFERENCES

(1) Ho, C.-H.; Lee, S. SERS and DFT Investigation of the Adsorption Behavior of 4-Mercaptobenzoic Acid on Silver Colloids. *Colloids Surf., A* **2015**, *474*, 29–35.

(2) Nagashree, K. L.; Lavanya, R.; Kavitha, C.; Narayanan, N. S. V.; Sampath, S. Spontaneous Formation of Branched Nanochains from Room Temperature Molten Amides: Visible and near-IR Active, SERS Substrates for Non-Fluorescent and Fluorescent Analytes. *RSC Adv.* **2013**, *3*, 8356–8364.

(3) Capocefalo, A.; Mammucari, D.; Brasili, F.; Fasolato, C.; Bordi, F.; Postorino, P.; Domenici, F. Exploring the Potentiality of a SERS-Active pH Nano-Biosensor. *Front. Chem.* **2019**, *7*.

(4) Williams, A.; Flynn, K. J.; Xia, Z.; Dunstan, P. R. Multivariate Spectral Analysis of pH SERS Probes for Improved Sensing Capabilities. *J. Raman Spectrosc.* **2016**, *47*, 819–827.

(5) Rosendahl, S. M.; Burgess, I. J. Electrochemical and Infrared Spectroscopy Studies of 4-Mercaptobenzoic Acid SAMs on Gold Surfaces. *Electrochim. Acta* **2008**, *53*, 6759–6767.

(6) Bishnoi, S. W.; Rozell, C. J.; Levin, C. S.; Gheith, M. K.; Johnson, B. R.; Johnson, D. H.; Halas, N. J. All-Optical Nanoscale pH Meter. *Nano Lett.* **2006**, *6*, 1687–1692.

(7) Wilson, E. B. The Normal Modes and Frequencies of Vibration of the Regular Plane Hexagon Model of the Benzene Molecule. *Phys. Rev.* **1934**, *45*, 706–714.

(8) Andrejeva, A.; Gardner, A. M.; Tuttle, W. D.; Wright, T. G. Consistent Assignment of the Vibrations of Symmetric and Asymmetric Para-Disubstituted Benzene Molecules. *J. Mol. Spectrosc.* **2016**, *321*, 28–49.

(9) Michota, A.; Bukowska, J. Surface-Enhanced Raman Scattering (SERS) of 4-Mercaptobenzoic Acid on Silver and Gold Substrates. *J. Raman Spectrosc.* **2003**, *34*, 21–25.

(10) Kwon, Y. J.; Son, D. H.; Ahn, S. J.; Kim, M. S.; Kim, K. Vibrational Spectroscopic Investigation of Benzoic Acid Adsorbed on Silver. *J. Phys. Chem.* **1994**, *98*, 8481–8487.

(11) Gardner, A. M.; Wright, T. G. Consistent Assignment of the Vibrations of Monosubstituted Benzenes. *J. Chem. Phys.* **2011**, *135*, 114305.

(12) Mulliken, R. S. Report on Notation for the Spectra of Polyatomic Molecules. *J. Chem. Phys.* **1955**, *23*, 1997–2011.

(13) Abdelsalam, M. Surface Enhanced Raman Scattering of Aromatic Thiols Adsorbed on Nanostructured Gold Surfaces. *Open Chem.* **2009**, *7*, 446–453.

(14) Kim, J.-H.; Twaddle, K. M.; Cermak, L. M.; Jang, W.; Yun, J.; Byun, H. Photothermal Heating Property of Gold Nanoparticle Loaded Substrates and Their SERS Response. *Colloids Surf., A* **2016**, *498*, 20–29.

(15) Ren, X.; Tan, E.; Lang, X.; You, T.; Jiang, L.; Zhang, H.; Yin, P.; Guo, L. Observing Reduction of 4-Nitrobenzenthiol on Gold Nanoparticles in Situ Using Surface-Enhanced Raman Spectroscopy. *Phys. Chem. Chem. Phys.* **2013**, *15*, 14196–14201.

(16) Skadtchenko, B. O.; Aroca, R. Surface-Enhanced Raman Scattering of p-Nitrothiophenol: Molecular Vibrations of Its Silver Salt and the Surface Complex Formed on Silver Islands and Colloids. *Spectrochim. Acta, Part A* **2001**, *57*, 1009–1016.
